# Self-report measures of subjective time: An overview of existing measures and their semantic similarities

**DOI:** 10.3758/s13423-026-02987-4

**Published:** 2026-08-25

**Authors:** Thiago Augusto de Souza Bonifácio, Rodrigo Cabral-Carvalho, André Mascioli Cravo

**Affiliations:** 1 Graduate Program in Neuroscience and Cognition, Federal University of ABC (UFABC), São Bernardo do Campo, Brazil; 2 Center for Mathematics, Computing and Cognition, Federal University of ABC (UFABC), São Bernardo Do Campo, Brazil

**Keywords:** Automated semantic analysis, Scale overlap, Semantic similarity, Subjective time

## Abstract

The proliferation of self-report measures in psychology, often developed without systematic differentiation from existing tools, raises concerns about construct redundancy and the interpretability of research findings. This study reviews self-report scales designed to assess subjective time, combining a scoping literature analysis with embeddings-based automated semantic analysis to map relationships among them. Thirty scales met inclusion criteria, drawn from experimental psychology, personality research, and organizational behavior. Hierarchical clustering revealed two broad groupings: Temporal Experience, which covers how individuals perceive, feel, and conceptualize time, and Time Perspective, which covers orientations toward the past, present, and future temporal frames. This two-cluster solution partially maps onto previous frameworks of subjective time but diverges in ways that reflect a recurring gap between theoretically motivated distinctions and how researchers operationalize constructs in items. Within the Time Perspective cluster, several Future Time Perspective instruments showed high semantic overlap without a principled account of their differences, suggesting a possible case of micro-level redundancy. Discrepancies between construct definitions and scale content were also identified across both clusters, with direct implications for measurement validity. A post hoc validation study found that model similarity scores correlated with human semantic judgments, supporting the method as a viable first-pass mapping tool. Embedding-based analysis can flag redundancy, but domain expertise remains necessary to interpret findings and draw theoretical conclusions.

## Background

Time pervades several aspects of human activities, organization, and individual experiences. However, while *objective time* (i.e., clock time) structures our world, our *perception of time* can be modulated by several factors. This *subjective time* is a multidimensional process involving an intricate interplay of interdependent abilities, such as memory, attention, and emotion (Kent et al., 2023; Pöppel, 1997; Vogel et al., 2020; Wittmann, 2009). Thönes and Stocker (2019) proposed a theoretical framework that identifies three core components of subjective time: (a) *temporal processing*, the ability to detect fundamental temporal information, such as the order and simultaneity of stimuli (Coull & Giersch, 2022); (b) *time perception in terms of passage*, the representation of events across past, present, and future, including their significance for guiding behavior (Chen & Zhao, 2024) and the subjective assessment of time’s perceived speed (Lamprou-Kokolaki et al., 2024; Martinelli & Droit-Volet, 2022); and (c) *time perception in terms of duration*, the felt experience of temporal extension, distinguishable from what objective measures (e.g., timers and clocks) record (Bueno et al., 2024).

These components have been studied extensively in experimental psychology and neuroscience, primarily through behavioral tasks (for a review, see Grondin, 2010). Most of this work has focused on components (a) and (c) in controlled settings, examining how people perceive the temporal structure of sensory events and what factors influence their interval estimates (Cui et al., 2023; Matthews & Meck, 2016). Controlled experiments offer precision, but they tend to foreground automatic and non-declarative processes at the expense of subjective time’s conscious and experiential dimensions (Flaherty, 2011). As Martinelli and Droit-Volet (2022) argue, equating temporal experience with its non-declarative aspects is inadequate. Subjective time is also shaped by conscious thoughts, emotions, cultural values, and interpretive biases (Casasanto & Bottini, 2014; Lake et al., 2016; Sinha, 2022). This is what makes self-report measures (i.e., standardized scales, surveys, and questionnaires; Corneille & Gawronski, 2024) a necessary complement.

Self-report instruments operate at the declarative level, capturing how individuals consciously represent, evaluate, and reflect on their relationship with time. Passage-of-time judgments, for instance, ask directly how fast or slow time feels relative to normal, using Likert-type (e.g., “very slow” to “very fast”) or visual analog scales (Wearden, 2015). Time-perspective instruments, in turn, ask how individuals organize and evaluate their experience across past, present, and future. Here, the construct is typically conceived as a stable individual difference, analogous to personality traits studied in psychology, that shapes goal-directed behavior, decision making, and well-being (Stolarski et al., 2018; Zimbardo & Boyd, 1999).

The declarative nature of self-report measures also makes them well suited to real-world contexts where behavioral tasks fall short. In a longitudinal study during the COVID-19 pandemic, temporal production tasks, self-report measures of time expansion (i.e., the feeling of time being dilated), and time pressure (i.e., the sense that available time is insufficient to meet one’s demands) varied independently across weeks of social distancing (Cravo et al., 2022). Time pressure (or time poverty, in social sciences literature) has also been linked to societal (e.g., acceleration of daily life), organizational (e.g., changes in work structure), institutional (e.g., bureaucratic time burdens), and psychological factors (e.g., undervaluation of time as a resource, psychological distress) in ways that laboratory tasks cannot easily address (Giurge et al., 2020; Rudd, 2019). When combined with specific designs (e.g., intensive longitudinal data) and instruments (e.g., wearable sensors), self-report measures can test whether mechanisms identified in the lab hold in daily experience and whether the variables that modulate them do so with the same strength outside controlled conditions (Ogden et al., 2022). The fact that these two approaches complement rather than substitute for one another makes the careful development and evaluation of subjective time scales a shared methodological priority.

Self-report instruments are now the principal tools for assessing psychological constructs across the behavioral sciences (Uher, 2018), but the instruments used to operationalize any given construct frequently vary across researchers (Maul, 2013), raising questions about comparability and knowledge accumulation. What appears to be a diverse measurement landscape may, on closer inspection, conceal substantial redundancy. Flake and Fried (2020) documented a widespread pattern: measures created and adopted without sufficient evidence of systematic development or differentiation from existing tools. This reflects, in part, the problem of construct redundancy, i.e., the tendency for new research to be organized around constructs indistinguishable from existing ones (Hodson, 2021). When nominally distinct constructs share similar items, correlations between scales become difficult to interpret, theoretical conclusions grow ambiguous, and instrument selection becomes opaque (Lawson & Robins, 2021).

Whether subjective time research is vulnerable to these problems remains unknown. The field spans experimental psychology, clinical research, organizational behavior, and cross-cultural studies, with distinct disciplinary communities employing instruments with limited coordination among them. This lack of coordination can have practical consequences: researchers may select redundant instruments, interpret correlations between semantically overlapping scales as theoretically meaningful, or pass over well-validated measures in favor of newer ones with weaker foundations. Addressing this requires both a systematic mapping of available instruments and methods capable of quantifying the overlap among them.

This study pursued two objectives: mapping existing self-report measures across its distinct dimensions, and characterizing their underlying constructs, definitions, and psychometric properties. Our approach draws on scoping review guidelines, which aim to provide a broad, exploratory overview of key concepts, research methods, and findings in a given area (Munn et al., 2022). To complement the qualitative analysis, we used Natural Language Processing (NLP) to quantify semantic similarity among the selected scales (for a comparable procedure, see Rosenbusch et al., 2020). Specifically, we used the Sentence-T5 (ST5) model, a sentence-embedding model from the Sentence Transformers family fine-tuned for similarity tasks (Ni et al., 2021), to generate embeddings of scale items and construct definitions, and then applied clustering techniques to group scales by semantic similarity.

The paper proceeds as follows. The next section describes the search strategy, eligibility criteria, data extraction, and NLP methodology. This is followed by sections presenting (a) the qualitative and semantic similarity results and (b) a human benchmark validation study. A final section discusses the findings, evaluates the method, and offers practical guidance for researchers selecting instruments in the subjective time domain.

## Material and methods

### Database search strategy and eligibility criteria

The literature search targeted peer-reviewed journal articles and used the following databases: PsycNET, PubMed, Scopus, and Web of Science. The search terms or keywords used were: (“time perception” OR “subjective time” OR “temporal experience” OR “time production” OR “time estimation” OR “time passage” OR “temporal orientation” OR “time perspective” OR “time attitudes” OR “time orientation” OR “time relation” OR “time–frequency”) AND (scale OR questionnaire OR inventory OR self-report OR measure) AND (development OR validation OR “psychometric properties”).

We established comprehensive eligibility criteria to identify relevant studies focusing on self-report measures with detailed psychometric information. The eligibility criteria were as follows: (1) we specifically focused on structured self-report measures, encompassing both formative and reflective types (Chang et al., 2016); (2) only multi-item scales were included, excluding single-item measures such as the commonly used to measure passage of time judgments (e.g., Martinelli & Droit-Volet, 2023; Tanaka & Yotsumoto, 2017), ensuring sufficient depth and breadth of psychometric information; (3) eligible papers that reported at least one reliability index, such as test–retest reliability or internal consistency; (4) we sought validity evidence based on test content, response processes, internal structure, or relations to other variables; (5) we limited our selection to papers published in English, with the stipulation that an English version of the scale must be presented within the articles, even if the scale itself was not initially developed in this language; (6) lastly, we restricted our search to scales published in peer-reviewed journals, with a specific emphasis on validation papers – for instance, the Temporal Metacognition Scale (Stolarski & Witowska, 2017), published in a book chapter, was not included due to its limited accessibility and the absence of scale items in the only study reporting its use (Witowska et al., 2022).

Backward and forward searches were applied to the included papers to identify additional relevant sources. This process was repeated iteratively until no relevant additional papers were found. While the literature search was last updated on 30 September 2023, a review protocol was not registered beforehand.

### Data extraction and descriptive analysis

The descriptive variables extracted were: (a) first author name; (b) publication year; (c) journal name; (d) sample size; (e) scale name; (f) construct definition; (g) number of items; (h) item content; (i) how responses are measured (i.e., type of response scale); (j) source of validity evidence (e.g., evidence based on test content, response processes, internal structure, or relations to other variables); and (k) reliability indicators (e.g., test–retest reliability, Cronbach’s alpha, McDonald’s omega, Composite Reliability). A complete data description is available on the Open Science Framework (OSF) (https://osf.io/rj3kx/).

### Semantic similarity analysis

All data analyses were performed using Python 3.12. Semantic similarity analysis was conducted using the SentenceTransformers framework v.2.2.2 (Reimers & Gurevych, 2019), while hierarchical clustering was performed using the SciPy library v1.11.2 (Virtanen et al., 2020). The project and detailed scripts for these analyses are available on GitHub (https://github.com/Rodrigo-Motta/Timing_LLM).

#### Generation of embeddings

Our primary approach involved calculating the cosine similarity between the vector representations (embeddings) of each target scale (“A”) and the other scales (“B”). We employed the Sentence-T5 (ST5) model to generate these embeddings. The ST5 is a sentence-embedding model from the Sentence Transformers family, fine-tuned for sentence-similarity tasks. It excels at producing high-quality sentence embeddings suitable for semantic retrieval and similarity assessment (Ni et al., 2021). Its encoder-decoder architecture maps sentences and paragraphs into a 768-dimensional dense vector space, capturing their semantic meaning and outperforming other models (e.g., Sentence-BERT and SimCSE) on various benchmarks, such as the Massive Text Embedding Benchmark (Muennighoff et al., 2022). Additionally, we calculated the cosine similarity between the reported definitions of the constructs. This allowed us to assess the degree of similarity between ST5 judgments based on scale items and judgments based on the definitions of each latent construct.

For each scale, item texts were concatenated into a single string, in the item order described below, and this string was submitted to ST5 as a single input to produce one questionnaire-level embedding (rather than embedding items separately and averaging their individual embeddings).

**Fig. 1:**
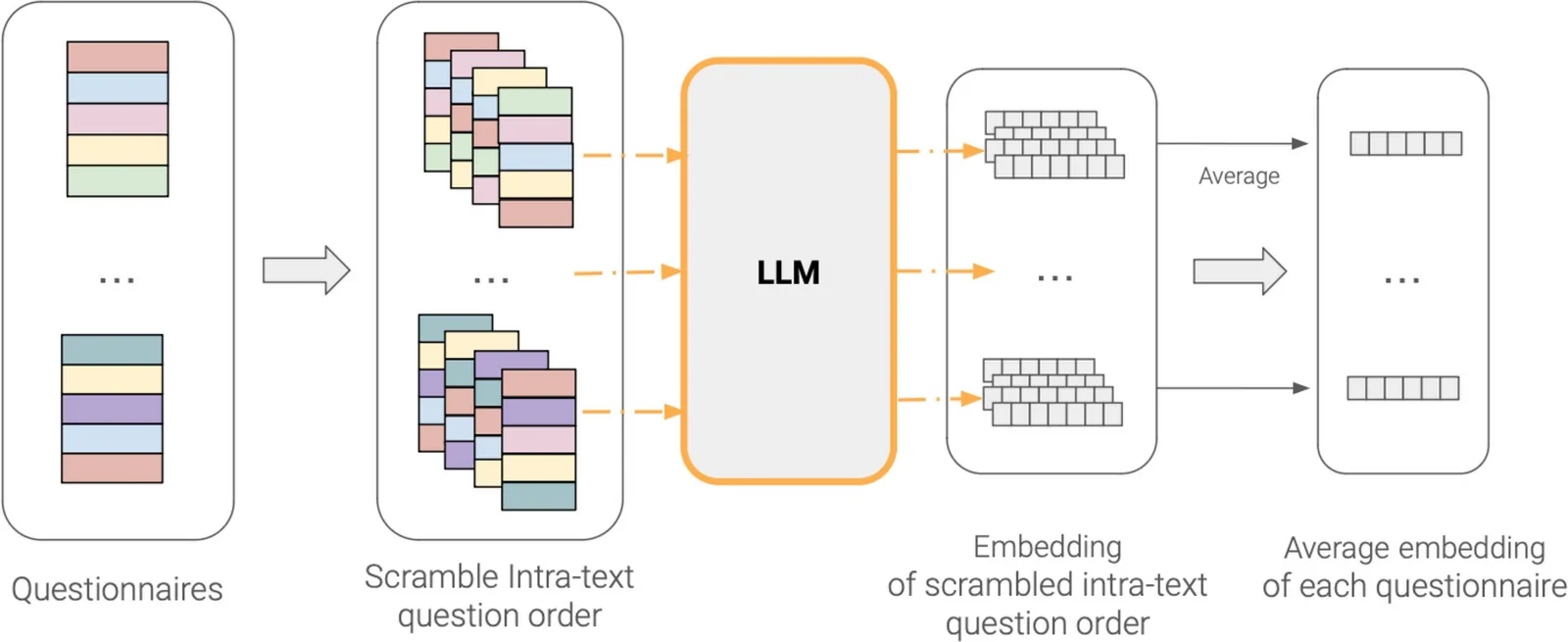
Illustration of the embedding generation process. Colored blocks represent each questionnaire, and the scrambling process is visualized. The final averaged embedding is shown as a single block

To ensure robustness against question order, each questionnaire’s questions were randomly scrambled 50 times. ST5 processed each scrambled version, generating 768-dimensional embeddings that encapsulate the semantic content regardless of question order. These embeddings were averaged to produce each questionnaire’s final, order-agnostic representation. Figure 1 illustrates the embedding generation process, where colored blocks represent each questionnaire. The scrambling process is visualized, culminating in a single, averaged embedding that represents the questionnaire content without being influenced by the specific order of questions.

We evaluated the standard deviation for each dimension to assess the impact of question order on the embeddings. The results indicate that question order had minimal influence on the embeddings, with the standard deviation for each dimension ranging from 1 to 10% of the mean value. This suggests that ST5 primarily captures semantic nuances rather than temporal patterns related to question order.

Because ST5 accepts at most 512 tokens, we checked the tokenized length of each scale’s concatenated string for truncation. Twenty-eight of the 30 scales fell within this limit while two did not: the Zimbardo Time Perspective Inventory (ZTPI; Zimbardo & Boyd, 1999; 807 tokens) and the Time Metaphors Questionnaire (Sobol-Kwapinska & Nosal, 2009; 723 tokens). For these two scales, any single encoding passes the cut-off part of the item content. We then tested whether the scrambling-and-averaging procedure described above offsets this loss. For each item on the scale, we tracked how often it remained intact across the 50 scrambled repetitions before reaching the token limit, and repeated this 1,000 times to assess whether the estimate held up. Inclusion rates were nearly identical across items and stable across repetitions (ZTPI: 62–63.1%, *M* = 62.5%; Time Metaphors Questionnaire: 69.8–70.8%, *M* = 70.3%), and no item was dropped from every scramble. Thus, each individual embedding for these two scales is built from incomplete input, but no single item is being systematically left out (i.e., the averaged embedding still draws on all of them).

#### Dimensionality reduction and exploratory clustering

To visualize potential clusters of questionnaires, we performed Principal Component Analysis (PCA) on the 768-dimensional embedding space. This allows easier visualization and interpretation of relationships among questionnaires. Following PCA, we performed k-means clustering on the embeddings. This algorithm partitions the data into *k* clusters by iteratively assigning each embedding to the nearest cluster center and updating the cluster centers based on the mean position of the embeddings assigned to each cluster (Ikotun et al., 2023). The k-means algorithm was initialized using the “k-means +  + ” method to improve cluster quality and was set to run for a maximum of 300 iterations, with ten different initializations to ensure stable clustering results. To evaluate the quality of the achieved solution, we computed the Silhouette Score, which measures how similar a data point is to its own cluster (cohesion) compared to other clusters (separation) (Rousseeuw, 1987).

#### Hierarchical clustering

Once the average embeddings for each questionnaire were calculated, a hierarchical clustering technique was employed using the nearest-neighbor chain (Müllner, 2011). This method estimates cluster distances using cosine distance as the inner product of the vector space, a similarity metric commonly used in semantic embedding models that efficiently estimates similarities in word representations in vector space (Mikolov et al., 2013). Hierarchical clustering groups questionnaires based on their pairwise distances, with the average linkage method chosen as it incorporates information about the variance of distances between clusters (Yim & Ramdeen, 2015). This approach groups self-report measures with similar semantic content, facilitating the identification of potential redundancies and overlaps in their underlying constructs.

#### Construct-item alignment analysis

To evaluate the alignment between construct definitions and scale content, we employed two strategies: (i) calculating the Spearman correlation between the average similarity matrices for construct definitions and scale content, with significance tested via 5,000 permutations (Kriegeskorte et al., 2008), and (ii) generating a new similarity matrix (construct definitions × scale content) using cosine similarity, followed by row-wise min–max normalization. From this normalized matrix, we created binary similarity matrices by selecting the top-*n* highest similarity values for each row, with *n* iteratively varied. The accuracy of construct-item matches was then computed as the percentage of correctly identified matches along the diagonal of the binary similarity matrix. This process was repeated for varying values of *n*, and accuracy percentages were recorded. Finally, we plotted the percentage of correctly matched scales against the corresponding *n* values.

## Results

Results are organized in two parts. The first two sections provide a qualitative overview of the 30 identified scales, including their psychometric properties and the constructs they assess. The third section reports the semantic similarity analyses, including an exploratory PCA and k-means visualization, a parallel analysis of construct definitions, pairwise similarity matrices and construct-item alignment, and hierarchical clustering. Each step builds on the previous: broad groupings first, then alignment between theoretical intent and item content, then pairwise relationships among all 30 scales.

### Overview of identified scales

**Fig. 2:**
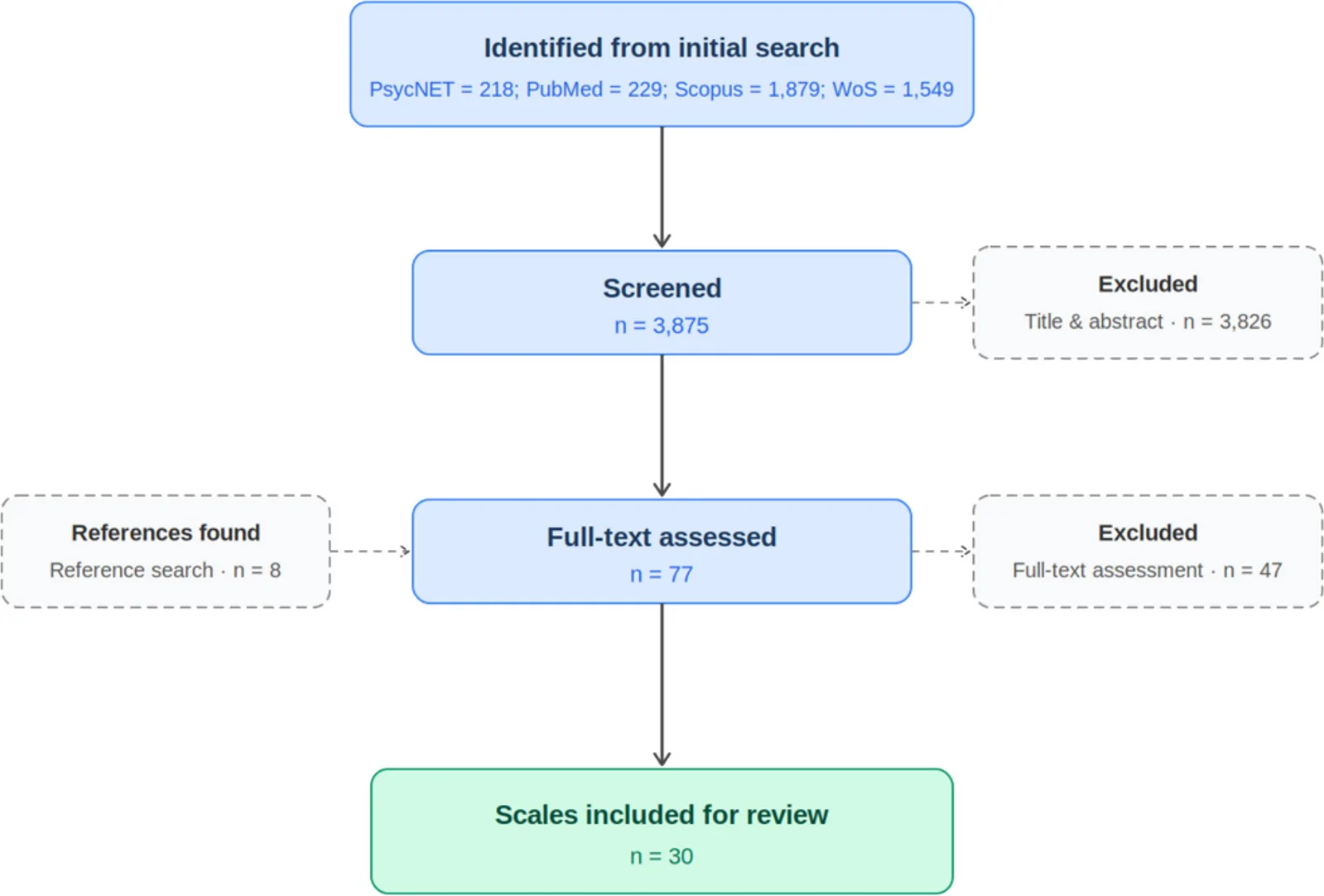
Study selection flowchart. Of the 3,875 identified records, 30 scales met all inclusion and exclusion criteria and were retained for qualitative synthesis and semantic similarity analysis

The literature search initially yielded 3,875 records. After removing duplicates and screening for eligibility, 77 full-text publications were reviewed, of which 30 scales met all inclusion criteria. The study selection process is illustrated in Fig. 2.

The 30 scales used Likert-type response formats with four to seven options, averaging 21 items per scale (range: 5–95), and were published between 1994 and 2023. Validation sample sizes ranged from 72 to 2,155 participants. The internal structure was predominantly assessed using exploratory and confirmatory factor analyses. Internal consistency was satisfactory in 90% of scales (*n* = 27; minimum α > 0.60; Bagozzi & Yi, 1988); three scales fell below this threshold (Lamotte et al., 2014; Rojas-Méndez et al., 2002; Wittmann & Lehnhoff, 2005).

Validity evidence was examined through relationships with different external measures, such as personality inventories (e.g., Big Five, NEO-PI, Five-Factor Inventory), well-being measures (e.g., depression scales, PANAS, Satisfaction with Life Scale), and other time-related instruments (e.g., Zimbardo Time Perspective Inventory, future time perspective scales). Additional correlates included locus of control, anxiety, rumination, stress, delay gratification, and self-esteem. The external measures used varied across studies; three did not report their use (Lukwago et al., 2001; Mello et al., 2016; Rojas-Méndez et al., 2002).

### Assessed constructs

Table 1 summarizes the 30 selected scales. The majority (*n* = 20) were developed to assess Time Perspective and related constructs, including Balanced Time Perspective (Webster, 2011), Time Attitudes (Mello et al., 2016; Rojas-Méndez et al., 2002; Sobol-Kwapinska & Nosal, 2009), Temporal and Time Orientation (Holman & Silver, 1998; Lukwago et al., 2001; Şimşek & Kocayörük, 2013; Sobol-Kwapinska, 2009), and Temporal Focus (Shipp et al., 2009). Within this group, particular attention has been devoted to the extent to which individuals consider distant outcomes when making decisions (i.e., Future Time Perspective; FTP), which is the focus of seven instruments (Biondolillo & Epstein, 2021; Boyd & Zimbardo, 1997; Carstensen & Lang, 1996; Lyu & Huang, 2016; Rutten et al., 2022; Strathman et al., 1994; Zaleski, 1996).

**Table 1:** Summary of selected scales, assessed constructs, and dimensions for each scale, ordered by publication date

ID	Scale name (abbreviation; authors, year)	Construct	Dimensions	Example item
22	**Consideration of Future Consequences Scale** (CFC-14; Strathman et al., 1994)	Consideration of Future Consequences	*Unidimensional*	My behavior is only influenced by the immediate outcomes of my actions
23	**Time Styles Scale** (Time-Styles Scale; Usunier & Valette-Florence, 1994)	Time Styles	Preference for Organized Time	I sometimes find myself dwelling in the past
			Time Submissiveness	
			Orientation Towards the Past	
			Orientation Towards the Future	
			Time Anxiety	
			Preference for Unorganized Time	
4	**Future Time Perspective Scale** (FTP Scale; Carstensen & Lang, 1996)	Future Time Perspective	*Unidimensional*	Most of my life lies ahead of me
28	**Future Anxiety Scale** (Zaleski, 1996)	Future Anxiety	*Unidimensional*	I am afraid to plan for the future
2	**Transcendental-FTP Inventory** (TTPI; Boyd & Zimbardo, 1997)	Transcendental-Future Time Perspective	*Unidimensional*	I will be held accountable for my actions on earth when I die
6	**Temporal Orientation Scale** (TOS; Holman & Silver, 1998)	Temporal Orientation	Past Orientation	I often talk about my past experiences with others
			Present Orientation	
			Future Orientation	
7	**Temporal Disintegration Scale** (TDS; Holman & Silver, 1998)	Temporal Disintegration	*Unidimensional*	In the last 24 h, how often did you feel as though time had stopped?
14	**Temporal Satisfaction with Life Scale** (TSLS; Pavot et al., 1998)	Temporal Satisfaction	Past Satisfaction	My life in the past was ideal for me
			Present Satisfaction	
			Future Satisfaction	
30	**Zimbardo Time Perspective Inventory** (ZTPI; Zimbardo & Boyd, 1999)	Time Perspective	Past Negative	Happy memories of good times spring readily to mind
			Past Positive	
			Present Hedonistic	
			Present Fatalistic	
			Future	
9	**Present and Future Orientation Scales** (PFOS; Lukwago et al., 2001)	Time Orientation	Present Orientation	What happens to me in the future is out of my control
			Future Orientation	
15	**Time Attitude Scale** (TAS; Rojas-Méndez et al., 2002)	Time Attitudes	Past Orientation	Children should be taught well the traditions of the past
			Present Orientation	
			Future Orientation	
			Successional Perspective	
			Time Pressure	
26	**Subjective Time Questionnaire** (STQ; Wittmann & Lehnhoff, 2005)	Time Awareness	Time Expansion	I often think time is running out
			Time Pressure	
			Metaphors, Speed	
			Metaphors, Slowness	
18	**Temporal Focus Scale** (TFS; Shipp et al., 2009)	Temporal Focus	Past Focus	I replay memories of the past in my mind
			Current Focus	
			Future Focus	
20	**Present Time Orientation Scale** (PTOS; Sobol-Kwapinska, 2009)	Present Time Orientation	Carpe Diem	It is worthwhile focusing on the present
			Fatalism	
			Hedonism	
21	**Time Metaphors Questionnaire** (TMQ; Sobol-Kwapinska & Nosal, 2009)	Time Attitudes	Friendly Time	Time flies like an arrow
			Hostile Time	
			Rapid Passage of Time	
			Significance of the Moment	
			Wild Time	
			Subtle Time	
			Empty Time	
25	**The Balanced Time Perspective Scale** (BTPS; Webster, 2011)	Balanced Time Perspective	Past	Reviewing events from my past helps give my life meaning
			Future	
19	**The Ontological Well-Being Questionnaire** (OWBQ; Şimşek & Kocayörük, 2013)	Ontological Well-Being	Nothingness	When I look at the future of my life project, I feel hopeful
			Hope	
			Regret	
			Activation	
3	**Multidimensional Questionnaire of FTP** (MFTP; Brothers et al., 2014)	Future Time Perspective	Future as Open	I look forward to the future with hope and enthusiasm
			Future as Limited	
			Future as Ambiguous	
8	**Metacognitive Questionnaire on Time** (MQT; Lamotte et al., 2014)	Awareness of Time Distortions	Self, Emotion	When I am bored, I feel time passes more slowly
			Self, Attention	
			Others, Emotion	
			Others, Attention	
10	**FTP Scale for Adolescents and Young Adults** (FTP-AYA; Lyu & Huang, 2016)	Future Time Perspective	Future-Negative	I think I can accomplish many things in the future
			Future-Positive	
			Future-Confusion	
			Future-Perseverant	
			Future-Perspicuity	
			Future-Planning	
11	**Adolescent and Adult Time Attitudes Scale** (AATAS; Mello et al., 2016)	Time Attitudes	Past Positive	My past makes me sad
			Past Negative	
			Present Positive	
			Present Negative	
			Future Positive	
			Future Negative	
24	**Present-Eudaimonic Time Perspective Scale** (P-ETP; Vowinckel et al., 2017)	Present Time Perspective	*Unidimensional*	I feel connected to myself when I stay in the moment
5	**Chronic Time Pressure Inventory** (CTPI; Denovan & Dagnall, 2019)	Chronic Time Pressure	Cognitive Awareness of Time Shortage	I feel in control of how I spend my time
			Feeling Harried	
13	**Time Perception Scale** (TPS; Niiya, 2019)	Time as a Resource (Time Conceptualization)	Taking Time	I feel that I am giving away my time to others
			Nonzero-sum Time	
			Offering Time	
			Time Taken Away	
1	**Scrambled Sentences Task for FTO** (Biondolillo & Epstein, 2021)	Future Time Orientation	*Unidimensional*	I frequently plan before acting
12	**Metacognitive Temporal Coping Questionnaire** (MTCQ; Morgenroth et al., 2021)	Metacognitive Temporal Coping	Impermanence Focus	I focus on how my feelings about the event may change with time
			Present Centeredness	
			Positive Temporal Refocusing	
			Negative Temporal Contrasting	
29	**Temporal Sense Scale** (TSS; Zhang et al., 2022)	Trait Time Passage	*Unidimensional*	How time usually passes for you across every day?
16	**Work Prospection Scale** (Rutten et al., 2022)	Work Prospection	Cognitive Work Prospection	I was looking forward to the workdays ahead of me
			Negative Affective Work Prospection	
			Positive Affective Work Prospection	
17	**Time Management and Estimation Scale** (TiME; Schiros et al., 2023)	Temporal Processing	Temporal Self-Regulation	It takes a long time to get through the day
			Time Pressure	
			Orientation to Time	
			Subjective Experience of Time	
27	**Metacognitive Experience of Time Passing Scale** (METP; Yu et al., 2023)	Metacognitive Experience of Time Passing	Ruminative Experience of Time Passing	The sense of time passing makes me reflect on myself
			Emotional Experience of Time Passing	

The numerical values in the “ID” column serve as unique identifiers for each scale, which correspond to the labels used in the figures throughout the *Results* section

The remaining ten scales address distinct dimensions of subjective time experience. Four assess constructs related to time perception in terms of duration or passage, such as time pressure (Denovan & Dagnall, 2019; Schiros et al., 2023; Wittmann & Lehnhoff, 2005; Zhang et al., 2022). Three measure metacognitive aspects of time experience (Lamotte et al., 2014; Morgenroth et al., 2021; Yu et al., 2023), capturing individuals’ awareness and regulation of their own temporal perceptions. Three scales were classified as thematically distinct from either group: the Time Styles Scale (Usunier & Valette-Florence, 1994), the Temporal Disintegration Scale (Holman & Silver, 1998), and the Time Perception Scale (Niiya, 2019). Detailed descriptions of all 30 scales are provided in the OSF (https://osf.io/rj3kx/).

### Semantic similarity analysis

#### Exploratory visualization: PCA and k-means clustering

**Fig. 3:**
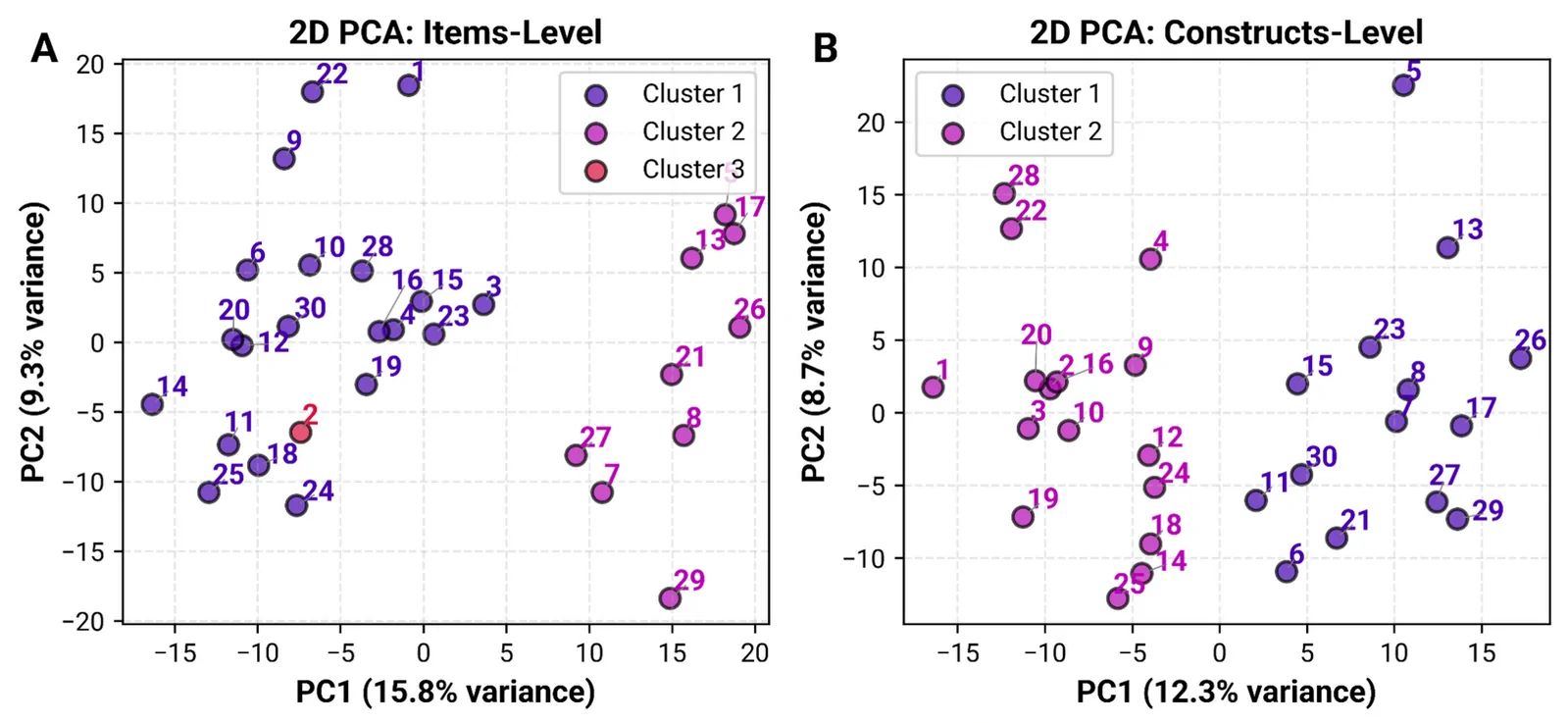
Two-dimensional PCA visualization of ST5 embeddings. (**A**) Embeddings derived from subjective time scale items. (**B**) Embeddings derived from construct definitions. In both panels, individual points represent specific scales, and colors indicate their respective k-means cluster assignments

Scale embeddings were reduced to 15 principal components via PCA, explaining 83.8% of the original variance, and submitted to k-means clustering, which identified two groupings and a singleton (Silhouette Score = 0.159; Fig. 3, Panel A). The resulting clusters aligned with our qualitative analysis. Cluster 1 (blue) comprised time perspective measures (*n* = 20), grouping both future-oriented scales and multidimensional inventories. Cluster 2 (purple) grouped scales directed at time perception in terms of duration or passage, including measures of time metaphors (Sobol-Kwapinska & Nosal, 2009), temporal metacognition (Lamotte et al., 2014), and time pressure (Denovan & Dagnall, 2019). The Transcendental-Future Time Perspective Inventory (TTPI; Boyd & Zimbardo, 1997) did not cluster with any other scale, indicating that its item content is sufficiently distinct from all other instruments to preclude meaningful cluster assignment. Although the TTPI appears near the other clusters in the two-dimensional (2D) visualization, in the full 15-dimensional embedding space, it is distant from both clusters. The low Silhouette Score suggests modest cluster cohesion, indicating that the boundaries between groups are not sharply defined. These results should therefore be interpreted with caution and combined with the hierarchical clustering solution reported in the section Hierarchical clustering, which captures more nuanced, pairwise relationships among the scales.

#### Construct-level analysis

A similar analysis was conducted on construct definitions rather than scale items. Construct definitions were extracted from each paper when available, or, when unavailable, inferred from the authors’ stated objectives (see Table S1 in Online Supplemental Materials on the OSF (https://osf.io/rj3kx/). Embeddings were generated, reduced via PCA, and submitted to k-means clustering (Fig. 3, Panel B).

Silhouette scores were low across all tested solutions (range: 0.04–0.08) and did not indicate a clear optimum, suggesting that the construct definitions do not partition into well-separated categories. The clustering serves as a visualization aid rather than a formal partitioning and the following interpretation emphasizes the relative positioning of scales in PCA space rather than discrete group membership.

The construct-level embedding space mirrored the item-level structure: future-oriented constructs occupied a coherent region, as did constructs related to time perception in terms of passage and duration. However, two divergences emerged. First, the TTPI (Boyd & Zimbardo, 1997), whose item content was distinct in the item-level solution, shifted toward the future-oriented construct region at the construct level, suggesting that its theoretical framing is less unusual than its items imply. Second, the ZTPI (Zimbardo & Boyd, 1999), positioned within the large time perspective cluster at the item level, moved toward the time perception region in PCA space, alongside instruments such as the Subjective Time Questionnaire (STQ; Wittmann & Lehnhoff, 2005) and the Metacognitive Questionnaire on Time (MQT; Lamotte et al., 2014). Constructs related to evaluative attitudes toward time and multidimensional time perspective were dispersed across the embedding space at both levels of analysis, indicating that item-level clustering does not reflect coherence at the construct definition level.

#### Pairwise similarity matrices and scale-construct alignment

**Fig. 4:**
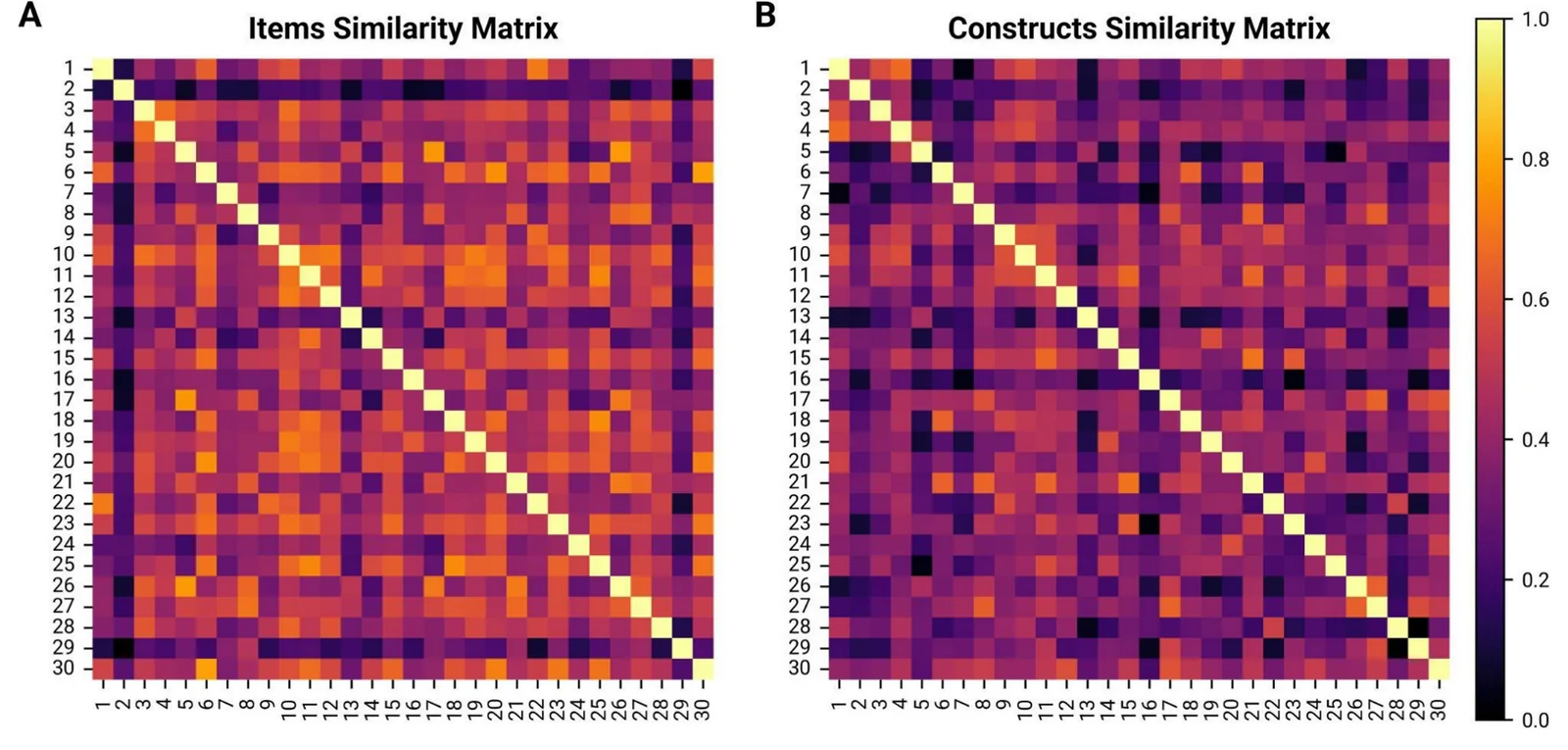
Item-level and construct-level cosine similarity matrices. (**A**) Item-level similarity matrix for all scale pairs. (**B**) Similarity matrix across construct definitions

**Fig. 5:**
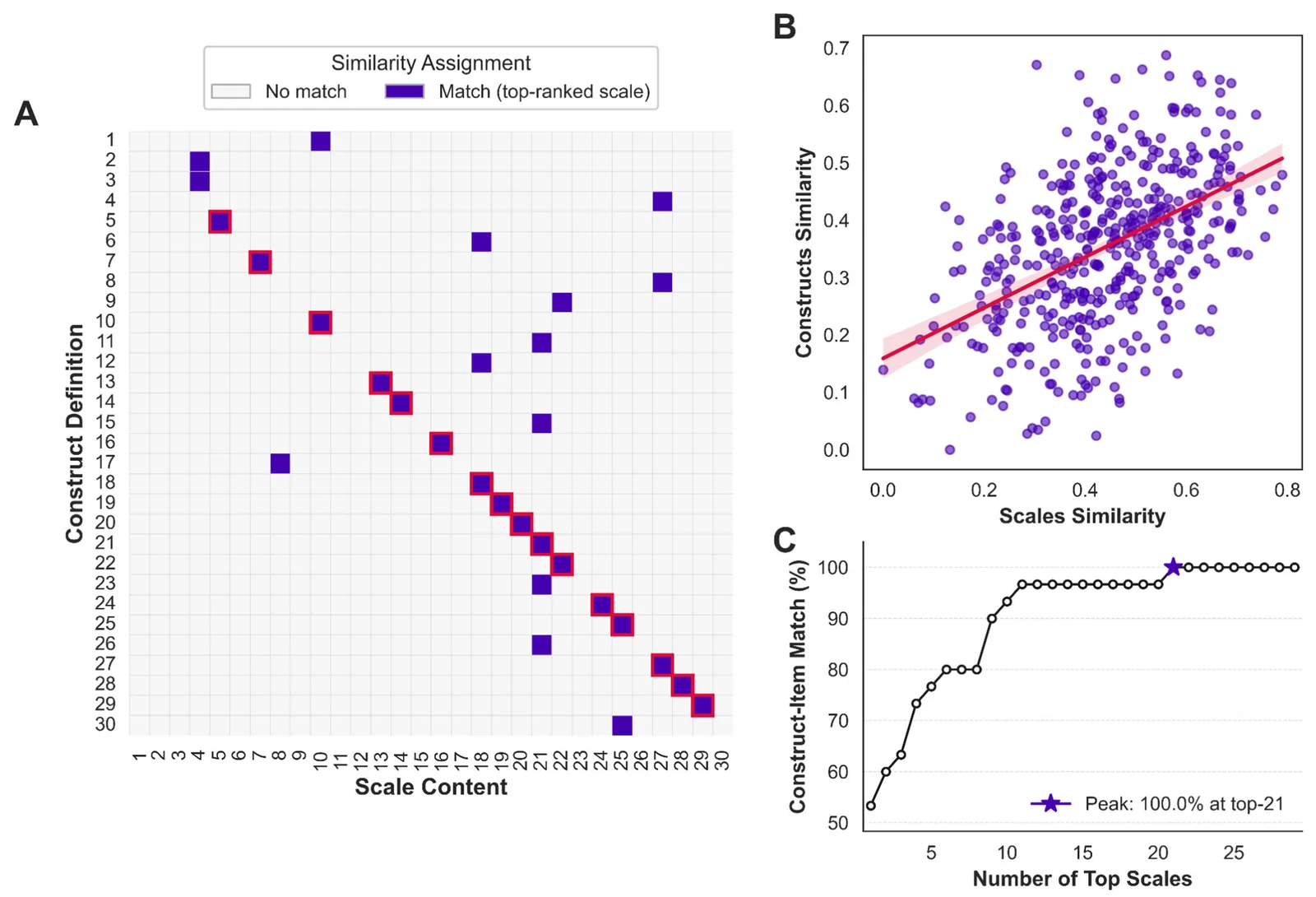
Construct-item matching accuracy between subjective time scales. (**A**) Binary similarity matrix of top-ranked scale assignments across constructs (*N* = 30). Purple cells indicate the single top-ranked scale for a given construct after row-wise min–max normalization; white cells indicate no assignment. Red outlines mark diagonal cells where construct and scale indices match (correct self-match), yielding a diagonal accuracy of 16/30 (53.3%). (**B**) Correlation between item-level and construct-level pairwise similarity scores. Each point represents a unique scale pair from the upper triangle of their respective similarity matrices. The red line indicates the fitted regression with a 95% confidence interval (ρ = 0.488). (**C**) Construct-item match accuracy as a function of the number of top-ranked scales considered. Each point represents the percentage of constructs whose matching scale appeared within the top-n ranked scales. The star marks the peak accuracy (100.0%) reached at top-21

Because item-level and construct-level capture different aspects of scale relationships (theoretical framing on one hand, operationalized content on the other), neither alone provides a complete picture. To quantify this, cosine similarity was computed for all scale pairs (Fig. 4, Panel A) and all construct-definition pairs (Fig. 4, Panel B), yielding two symmetric 30 × 30 matrices. To assess alignment between the two, we correlated the upper triangles of the two. The resulting moderate correlation (ρ = 0.488, p < 0.001) indicates only partial agreement between item-level and construct-level similarity; scales that are semantically similar at the item level are not always grounded in similarly defined constructs (Fig. 5, Panel B).

To quantify further this misalignment, we assessed whether each construct’s embedding was most similar to its own scale’s items across the full 30 × 30 construct-by-scale binary matrix (Fig. 5, Panel A). Only 16 of 30 constructs (53%) showed the highest similarity with their own scale. Expanding to the top two matches increased this to 60%, with accuracy plateauing at 21 scales (Fig. 5, Panel C). These analyses indicate that while a majority of constructs align with their instruments, a minority do not, and that item-level and construct-level similarity provide different information about scale relationships.

#### Hierarchical clustering

**Fig. 6:**
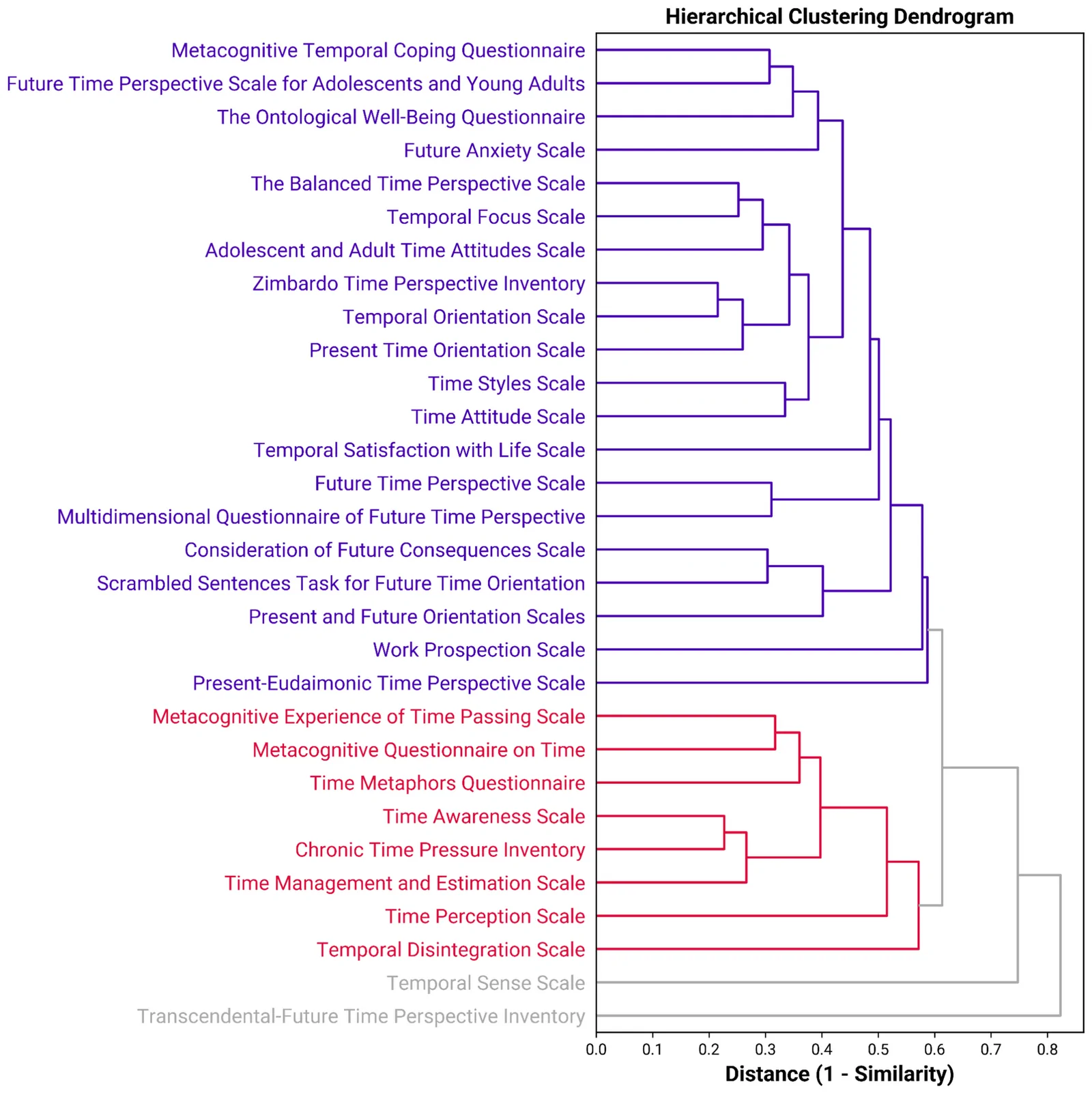
Dendrogram based on normalized cosine distances (1-similarity) between subjective time scales. Two major clusters are identified: Time Perspective (blue) and Temporal Experience (red). Two scales (gray) did not fit either cluster

Hierarchical clustering of the normalized scale-similarity matrix (Cophenetic Correlation Coefficient = 0.815) revealed two major clusters that closely replicated the initial k-means solution while providing finer-grained information on within-cluster relationships (Fig. 6).

The first cluster, labeled *Time Perspective*, grouped instruments that assess evaluative orientations toward the past, present, and future temporal horizons. The second, labeled *Temporal Experience*, comprised instruments focused on time conceptualization (Niiya, 2019; Sobol-Kwapinska & Nosal, 2009), temporal disintegration (Holman & Silver, 1998), time pressure and passage (Denovan & Dagnall, 2019; Schiros et al., 2023; Wittmann & Lehnhoff, 2005), and temporal metacognition (Lamotte et al., 2014; Yu et al., 2023). Two scales fell outside both clusters: the TTPI (Boyd & Zimbardo, 1997) and the Temporal Sense Scale (TSS; Zhang et al., 2022).

The highest pairwise similarity in the dataset (0.792) was between the Temporal Orientation Scale (TOS; Holman & Silver, 1998) and the ZTPI (Zimbardo & Boyd, 1999), which is unsurprising given that 13 of the 28 TOS items were drawn from the ZTPI (Holman & Silver, 1998, p. 1154). This finding validates the sensitivity of ST5 to item-level overlap.

The lowest pairwise similarity (0.056) was between the TTPI (Boyd & Zimbardo, 1997) and the Work Prospection Scale (Rutten et al., 2022), reflecting the conceptual distance between post-mortem transcendental belief (TTPI) and purposeful reflection on upcoming work tasks. This pair illustrates the breadth of the construct space captured by the 30 scales and anchors the lower bound of the similarity distribution.

Across all three analyses, the TTPI (Boyd & Zimbardo, 1997) showed a consistent pattern of divergence. At the item-level, it did not cluster with any other scale in the k-means solution and fell outside both clusters in the hierarchical solution. At the construct-level, however, it shifted toward the future-oriented region, suggesting that its construct definition shares enough vocabulary with established time perspective constructs to position it within that tradition at the definitional level, but its items fail to operationalize anything concrete within it. This produces a semantic profile that is recognizable in the abstract and anomalous in practice, illustrating how a poorly grounded construct can appear theoretically coherent while remaining empirically isolated, and why construct definitions and item content must be evaluated independently.

## Empirical study

To assess how well the ST5 model’s outputs align with human semantic judgment, we conducted a post hoc experiment in which participants provided pairwise similarity ratings on a subset of scale pairs.^1^

### Participants

Nineteen participants were recruited via convenience sampling (*M*_*age*_ = 25.16, *SD* = 5.28 years, range: 21–41 years; 42.1% women, 57.9% men). Inclusion criteria were age 18 years or older, willingness to participate with written informed consent, and self-reported English fluency of B1 or above. Scale items were presented in English to ensure judgments reflected the original constructs and mirrored the ST5 analysis, avoiding distortions introduced by translation. The study was approved by the Ethics Committee of the Federal University of ABC (approval number: 71017023.0.0000.5594).

### Materials and procedure

Data collection took place in person at the Human Cognition Laboratory of the Federal University of ABC; each session lasted approximately 25 min. Participants were tested individually. Before the task, they were informed of the study’s objectives, questions were addressed, and written informed consent was obtained. Participation was voluntary; participants were told they could withdraw at any time without consequence.

Six scales were selected to represent the cluster structure identified in the hierarchical clustering analysis. Pair selection followed two criteria: intra-cluster cohesion (the most semantically similar pairs within the same cluster, to validate cluster coherence) and inter-cluster distinction (pairs drawn from different clusters, to test whether the model’s between-cluster separability is reflected in human judgments). A practical constraint was also applied: selected scales had to contain a number of items feasible for simultaneous on-screen presentation. The scales were the TOS (Holman & Silver, 1998), the Present Time Orientation Scale (PTOS; Sobol-Kwapinska, 2009), the Time Management and Estimation Scale (TiME; Schiros et al., 2023), and the Chronic Time Pressure Inventory (CTPI; Denovan & Dagnall, 2019), plus two scales that fell outside the identified clusters: the TSS (Zhang et al., 2022) and the TTPI (Boyd & Zimbardo, 1997). The six scales yielded 15 unique pairwise combinations, each constituting one trial.

Participants first read written instructions explaining the task and completed three practice trials using scales not included in the main task. In both practice and main trials, the complete item sets for the two scales were displayed side by side on the screen. Participants evaluated the overall semantic similarity of the two item sets (as a whole, not item by item) on a Visual Analog Scale from 0 (“Completely different in meaning”) to 100 (“Completely identical in meaning”). Pair order and left/right screen positioning were randomized across participants.

### Data analysis

All analyses were conducted in R (version 4.4.3). Data were organized in long format (one observation per participant per scale pair) and merged with demographic information, including self-reported English fluency, treated as an ordered factor (B1 < B2 < C1 < C2). Human ratings were rescaled to the 0–1 range to enable direct comparison with ST5 cosine similarity scores.

#### Inter-rater reliability

Consensus among participants was assessed using two complementary approaches. First, a two-way average-measures ICC with absolute agreement was computed across the full 15-pairs × 19-raters matrix. Second, at a pairwise level, Spearman correlations were computed for all possible pairs of raters (each rater with all others, totaling 171 pairs), and their mean served as a complement to the ICC.

#### Human-model agreement

To test whether participants’ ratings were related to ST5 model scores, we estimated, for each participant, a Spearman correlation between the reported similarities and the model’s estimated similarities. At the group level, the coefficients were Fisher Z-transformed and submitted to a one-sample *t*-test against zero. The mean Z was back-transformed for interpretability. Lastly, to assess whether linguistic proficiency influenced this human-model agreement, participants’ self-reported English fluency levels were converted into a numeric ordinal scale (1 = B1 to 4 = C2), and a Spearman correlation was then calculated between each participant’s fluency level and their individual human-model agreement coefficient.

### Results

The validation study produced two main findings. First, inter-rater agreement was moderate on average, confirming that participants shared a broadly consistent understanding of semantic similarity despite some individual variability. Second, the human benchmark correlated positively with ST5 model scores, indicating good agreement between the two.

Most participants showed positive correlations with both the model scores and the group consensus. Two participants (3 and 14) showed near-zero or negative correlations on both indices; omitting them from the main analyses left the findings substantively unchanged.

**Fig. 7:**
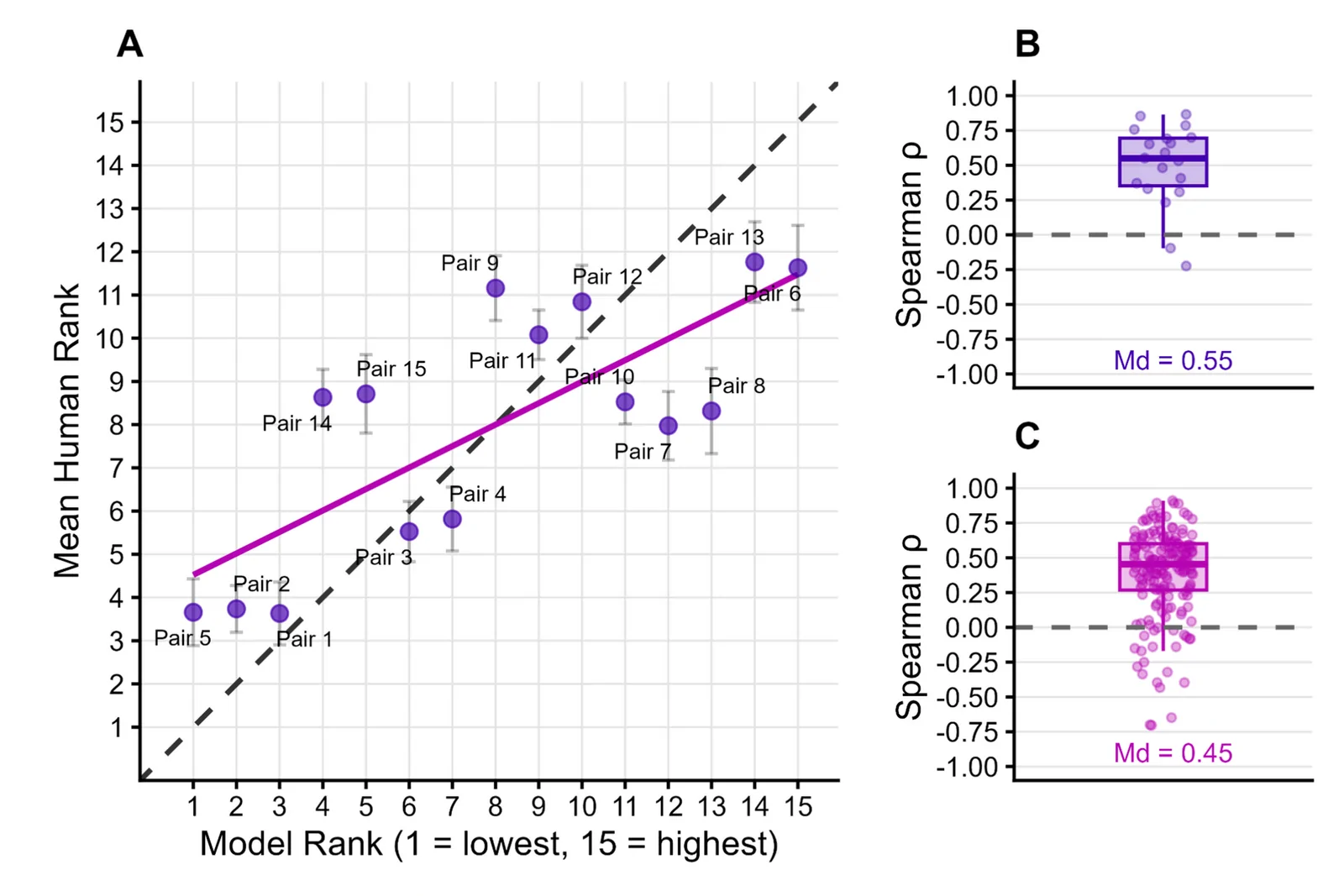
Agreement between human raters and the ST5 model. (**A**) Model vs. mean human ranks across 15 scale pairs. Dashed line = perfect agreement; solid line = fitted regression; error bars =  ± 1 SE. (**B**) Individual human–model Spearman correlations (*N* = 19; *Md* = 0.55). (**C**) All 171 pairwise inter-rater Spearman correlations (*Md* = 0.45). Dashed horizontal lines in B and C indicate ρ = 0

Individual ratings correlated positively with ST5 model scores on average (Fig. 7, Panel A): mean ρ (back-transformed) = 0.549, 95% CI [0.396, 0.672], *t*(18) = 6.54, *p* < 0.001, Cohen’s *d* = 1.50. Pairwise inter-rater correlations across all 171 rater pairs showed moderate average agreement (median ρ = 0.45, mean ρ = 0.39, *SD* = 0.32, range: − 0.70–0.91), with considerable variability reflecting individual differences in how people judge semantic similarity. The ICC showed a similar pattern, with good reliability (ICC(A,19) = 0.922, 95% CI [0.851, 0.969], *F*(14, 263) = 13.3, *p* < 0.001), indicating that mean human ratings can serve as a stable benchmark for model validation. Panels B and C of Fig. 7 show the distribution of individual human–model Spearman correlations and the 171 pairwise inter-rater correlations.

Furthermore, the analysis revealed no association between the raters’ English fluency and their agreement with the model similarities scores (ρ = 0.075, *p* = 0.759). This indicates that participants’ ability to assess semantic similarity in line with the algorithm was neither biased nor affected by their self-reported fluency.

## General discussion

This study mapped self-report measures of subjective time through a literature search, qualitative analysis, and embeddings-based semantic analysis. Thirty scales met our inclusion criteria, and clustering revealed two broad groupings (Temporal Experience and Time Perspective) whose structure, redundancies, and limitations we discuss below.

### Overview of identified scales

The 30 scales span three decades (1994–2023), range from five to 95 items, and were developed across experimental psychology, personality research, and organizational behavior. The actual number of subjective time scales in the literature is probably higher, as our inclusion criteria drew boundaries around a moving target. There is no straightforward criterion for determining whether thirty scales are too many or too few. What matters more than the count is the degree of similarity among them (Fried, 2017). Thirty genuinely distinct instruments serve a field well while 30 partial operationalizations of the same construct create interpretive problems that accumulate across studies.

### Cluster structure: Time perspective and temporal experience

Hierarchical clustering revealed two main clusters: Time Perspective and Temporal Experience. Before examining each, it is useful to situate them within the Thönes and Stocker (2019) framework introduced in the *Background* section. The Time Perspective cluster aligns with their time perception in terms of passage branch, which concerns how events are represented across temporal horizons and the significance of this representation in guiding behavior. The Temporal Experience cluster maps onto both the duration and passage branches, covering how individuals experience the speed and extension of time.

The observed two-cluster solution does not fit Thönes and Stocker’s tripartite taxonomy. This suggests that the way constructs are operationalized in scale content does not align with theoretical distinctions from experimental work. For example, scales measuring time pressure and temporal metacognition are theoretically closer to the constructs of duration and passage. Yet they cluster with instruments as diverse as time-metaphor use and temporal disintegration. What groups them is not theoretical proximity, but a shared first-person, experiential register which, in turn, experimental frameworks do not treat as a distinct category.

The following subsections examine each cluster in turn, describe the redundancies and misalignments identified within them, and discuss their implications for instrument selection.

#### The time perspective cluster

The Time Perspective cluster groups instruments that focus on how individuals orient toward and evaluate the past, present, and future temporal frames. The ZTPI (Zimbardo & Boyd, 1999) is widely considered the first standardized measure of Time Perspective and serves as the standard in the field. Therefore, it is unsurprising that it is grouped in this cluster and forms the highest similarity pair in our analysis, as several subsequent measures were explicitly inspired by it. Its high similarity to the TOS (Holman & Silver, 1998) and the Present Time Orientation Scale (PTOS; Sobol-Kwapinska, 2009) follows from that lineage: the TOS was partly built from ZTPI items, and the PTOS draws on the same conceptual vocabulary. Conversely, the Present-Eudaimonic Time Perspective Scale (P-ETP; Vowinckel et al., 2017) was a notable outlier. It focused on present-moment eudaimonic experience and remained distant from the other instruments, suggesting the model captures real construct-level differences within the cluster rather than surface-level groupings.

Construct boundaries blur at the item-level across much of this cluster. The Temporal Focus Scale (TFS; Shipp et al., 2009) clustered with the Balanced Time Perspective Scale (BTPS; Webster, 2011) and the Adolescent and Adult Time Attitudes Scale (AATAS; Mello et al., 2016) despite distinct framings. More striking is that the Zimbardo and Boyd (1999) construct definition showed greater similarity to the Temporal Focus Scale than to the ZTPI itself, a case in which the label signals one construct and the items reflect another, discussed further in the *Construct item alignment* section below.

The cluster also contains a concentration of Future Time Perspective (FTP) measures. FTP, the extent to which individuals consider distant outcomes when making decisions, is a central construct in several influential theoretical frameworks, including Socioemotional Selectivity Theory (Carstensen, 2021; Carstensen et al., 1999) and Temporal Construal Theory (Liberman & Trope, 1998; Trope & Liberman, 2003). We identified three FTP-labeled instruments (Brothers et al., 2014; Carstensen & Lang, 1996; Lyu & Huang, 2016) alongside several whose theoretical distinctiveness from FTP is underspecified, such as future anxiety (Zaleski, 1996), consideration of future consequences (Strathman et al., 1994), future time orientation (Biondolillo & Epstein, 2021), and work prospection (Rutten et al., 2022). This is the clearest case of micro-level redundancy in our data, taken up in the *Interpreting semantic redundancy* section below.

#### The temporal experience cluster

While the Time Perspective cluster is thematically focused, the Temporal Experience cluster is not. It includes measures of time as a social resource (Niiya, 2019; “*I feel that I am stealing other’s time*”), time metaphors (Sobol-Kwapinska & Nosal, 2009; “*Time is a voice of encouragement*”), temporal disintegration under trauma (Holman & Silver, 1998; “*In the last 24 h, how often did you feel as though time had stopped?*”), chronic time pressure (Denovan & Dagnall, 2019; “*There aren’t enough hours in the day*”), and temporal metacognition, that is, the awareness, understanding, and regulation of time-related thoughts and emotions (Lamotte et al., 2014; Yu et al., 2023; “*The sense of time passing makes me reflect on myself*”; “*When I am bored, I feel time passes more slowly*”). These instruments share a focus on how time feels in the immediate, first-person sense, not in terms of past-present-future orientation and goal-directed behavior.

Instruments in the Temporal Experience cluster tend to predict outcomes where the felt quality of time drives the effect (e.g., psychological distress symptoms, context-sensitive affect regulation) rather than outcomes tied to long-horizon planning or goal prioritization. The relevant criterion variables are themselves experiential and context dependent. Measures of attentional and emotional modulation of time passage have been linked to empathy (Lamotte et al., 2014); temporal disintegration scales are associated with trauma and dissociative disorders (Holman et al., 2023); and time pressure instruments are sensitive to societal, organizational, and institutional factors such as felt acceleration of daily life and changes in work structures (Giurge et al., 2020). Time Perspective instruments, in contrast, tend to predict dispositional and evaluative outcomes, such as life satisfaction, risk-taking behaviors, intertemporal choices, and self-regulation (Baird et al., 2021; Dou et al., 2023; Macaskill et al., 2019). The distinction matters for scale selection. A researcher studying how emergency responders cope with acute cognitive overload, for instance, needs a temporal experience instrument rather than a time perspective instrument, even though both concern “time.” Choosing among instruments within these clusters requires specifying the mechanism to be captured, a theoretical decision that precedes and constrains measurement, and which automated semantic analysis cannot resolve.

Within the Temporal Experience cluster, semantic grouping does not mean the instruments are necessarily redundant. Temporal disintegration under trauma is not interchangeable with chronic time pressure; time metaphor use is not interchangeable with metacognitive awareness of time passage. They share a phenomenological register but serve distinct measurement purposes. This raises the question about how to choose between related but not equivalent scales. We take this up in the sections that follow.

#### Interpreting semantic redundancy

The clustering results reduce the search space by grouping related instruments, flagging candidate pairs worth scrutinizing for redundancy, and revealing the field’s broad organizational structure. None of this entails that all scales within a cluster are interchangeable. Whether they are depends on the distinction between *semantic similarity*, a quantitative measure of shared linguistic content between two texts (Reilly et al., 2025), and *functional equivalence*, meaning interchangeability for a given research purpose.

Redundancy operates at two levels. First, the semantic overlap between instruments covering adjacent regions of a composite construct, what we call *macro-level redundancy*, is expected. Psychological constructs can be conceived as areas rather than points in a construct space: heterogeneous, hierarchically organized, and never fully captured by any single operationalization (De Boeck et al., 2023). Under this view, different instruments sampling overlapping but non-identical regions of the same construct space are not redundant in any damaging sense; they reflect the compositeness of the construct itself. A multidimensional inventory like the ZTPI (Zimbardo & Boyd, 1999) and a unidimensional measure like the CFC-14 (Strathman et al., 1994) share a semantic neighborhood because they draw on the same broad construct area; the former samples it more broadly, whereas the latter samples it more narrowly. In this sense, a researcher interested in future orientation might consider using the ZTPI Future subscale as an alternative to a dedicated FTP instrument. Although possible, the psychometric properties of that subscale were established within a multidimensional structure, and its behavior outside that context is not guaranteed to be equivalent. Its theoretical scope also differs from dedicated FTP instruments, capturing a general hedonic and planning orientation toward the future. In contrast, instruments like the Future Time Perspective Scale (FTP Scale; Carstensen & Lang, 1996) are anchored in the perception of a shrinking time horizon associated with aging. Using one as a proxy for the other conflates constructs that are semantically proximate but theoretically distinct.

The second level, *micro-level redundancy*, is more consequential. It occurs when instruments within the same sub-category (within a cluster) show high semantic overlap without a principled account of how they differ in theoretical terms or what they add in empirical terms. The proliferation of Future Time Perspective measures is the clearest case in our data. Three FTP-labeled instruments appear in our review (Brothers et al., 2014; Carstensen & Lang, 1996; Lyu & Huang, 2016), alongside several whose theoretical distinctiveness from FTP remains underspecified. The automated semantic analysis places these in proximity but without a principled account of how they differ, and without evidence of incremental validity over existing instruments, any selection risks being driven by convenience rather than construct alignment. This is the pattern Flake and Fried (2020) identified as systemic in psychology, in which measures are adopted without sufficient evidence that they differ from existing tools.

The time-pressure sub-cluster is a harder case and a useful illustration of what automated semantic analysis can and cannot do. The method identifies the TiME (Schiros et al., 2023), the CTPI (Denovan & Dagnall, 2019), and the STQ (Wittmann & Lehnhoff, 2005) as a zone of potential micro-level redundancy, which warrants closer inspection. That inspection, however, reveals a more nuanced picture. All three scales share a common core: items capturing the subjective experience of temporal scarcity and the affective response to it, that is, feeling rushed, pressured, and unable to complete what one sets out to do. This is the primary property that anchors all three to the Time Pressure construct. Where they diverge is in the secondary properties each instrument chose to operationalize around that core. The TiME (Schiros et al., 2023) extends into behavioral self-regulation and time-estimation accuracy; the STQ (Wittmann & Lehnhoff, 2005) incorporates metaphorical representations of time and the experience of time emptiness and boredom, dimensions absent from the other two scales; the CTPI (Denovan & Dagnall, 2019) remains closest to the affective-experiential core, with little extension beyond it. Under De Boeck et al.’s (2023) framework, this pattern is what one would expect from instruments sampling overlapping but non-identical regions of the same construct area. The semantic proximity is real and meaningful, but so are the differences. What the method cannot determine is how well each instrument’s particular combination of primary and secondary properties is justified and whether the extensions beyond the shared core reflect deliberate, well-grounded theoretical choices or accumulated item drift.

We propose that scale selection be anchored in four criteria: theoretical alignment between construct definition and operationalization, psychometric quality and validation evidence, practical cost, and appropriateness for the target population and research question. New instruments are only justified when their underlying construct is distinct from existing ones, a distinction that must be reflected in the items, not just in the definition, and when they demonstrate incremental validity over available alternatives. Where these conditions are not met, the field is better served by using existing instruments well and understanding their coverage limits than by adding to an already crowded measurement toolkit. That evaluation and the practical guidance it supports are discussed in the *Practical recommendations* section.

#### Construct-item alignment

A related issue is the alignment between a scale’s stated construct definition and its actual item content. Only 53% of constructs showed the highest similarity with their own scale, suggesting that construct-item misalignment is common across the field and not confined to a few ill-designed instruments.

The TTPI (Boyd & Zimbardo, 1997) is the clearest case. At the construct-definition level, the ST5 places it within the time perspective family, plausible given its nominal framing. At the item-level, it diverges from every other measure in the review. The construct-item alignment analysis confirmed weak correspondence between the TTPI’s stated definition and its items, consistent with criticisms by Seema et al. (2014), who argued the scale measures post-mortem belief systems rather than temporal cognition.

The gap between what a scale claims to measure and what its items actually ask is not unique to subjective time research but a recurring theme in measurement theory. Construct definitions tend toward the abstract and idealized. On the other hand, items are written under practical constraints and can drift from the original theoretical intent, sometimes reflecting specific assumptions that the definition never made explicit (Maul et al., 2016). De Boeck et al. (2023) argue that operational definitions, that is, item content, cover only parts of constructs, describing this as a structural feature of psychological measurement rather than a correctable flaw. In our view, automated semantic analysis can help by quantifying this gap through independent evaluation of construct definitions and item content, rather than taking a scale’s self-description at face value, and by flagging which instruments are most at risk of validity problems arising from this misalignment.

### Methodological considerations

How much confidence can we place in the ST5-based semantic map? We address this question by comparing the approach to existing alternatives, evaluating it against human judgments, and acknowledging its limitations.

#### Comparing the embeddings-based approach with other approaches

Automated semantic analysis methods for psychological scales have increased over the past decade. Rosenbusch et al. (2020) introduced the Semantic Scale Network, which detects overlap between scales using Latent Semantic Analysis (LSA). LSA decomposes a document-term matrix via singular value decomposition to recover latent dimensions of shared meaning, assuming that words co-occurring across texts reflect the same underlying topic. This extends basic word-matching techniques to latent structure. However, the estimates are still based on co-occurrence statistics in a fixed corpus, making LSA insensitive to context-dependent meanings and subtle linguistic nuances, such as the semantic relationships among homonyms, synonyms, or paraphrased expressions (Danish et al., 2024; Raiaan et al., 2024). For instance, “I feel rushed” and “I rarely have enough time” may mean the same thing, but LSA will treat them as unrelated.

Here, we used ST5, a pre-trained transformer-based sentence-embedding model fine-tuned for semantic-similarity tasks. Transformer architectures encode full sentences in context, capturing word-order dependencies and resolving meaning from surrounding words (Raiaan et al., 2024). The ST5 maps each scale into a 768-dimensional vector space where cosine similarity reflects semantic relatedness rather than lexical overlap (Ni et al., 2021). Wulff and Mata (2025) benchmarked LSA against several transformer models on predicting empirical correlations between personality items and five well-validated inventories. LSA’s predictive validity for internal consistency was near zero (*r* = 0.03); transformer models ranged from *r* = 0.40–0.75 in-sample and *r* = 0.40–0.61 out-of-sample. At the item-level, LSA recovered the factor structure of 13.5% of scales; fine-tuned MPNet recovered 100% in-sample and 79% out-of-sample. The gap held across convergent and divergent validity criteria.

The two approaches also differ in how their representational spaces are built. LSA’s latent space depends on the corpus fed into it; the Semantic Scale Network contained 4,037 scales at the time of publication, a large but bounded sample. Transformer models, by contrast, are pre-trained on broad general corpora and then fine-tuned, which allows them to generalize to semantic relationships absent from any specific scale collection (Raiaan et al., 2024).

Our study also differs from Rosenbusch et al. (2020) in scope. The Semantic Scale Network was designed to flag pairwise overlap between a new scale and an existing corpus. We applied the same underlying logic to a closed set of subjective time scales, then used hierarchical clustering over the full similarity matrix to map the structure of the entire domain, not just local redundancies. The construct-item alignment analysis, asking whether scale content matches theoretical definitions, is a question the Semantic Scale Network was not built to answer.

Our approach converges with Wulff and Mata (2025), who applied transformer embeddings to detect jingle-jangle fallacies in the International Personality Item Pool. Both projects use sentence embeddings and cosine similarity to map relationships among psychological measures. The methods differ in one important respect: Wulff and Mata worked with a well-validated personality corpus large enough to support fine-tuning on empirical correlation data. We were working in a domain with no comparable benchmarks, where the goal was exploratory, that is, mapping construct relationships across a heterogeneous set of scales built under divergent theoretical traditions. We used a general-purpose ST5 rather than a domain–fine-tuned model, and the fine-tuning approach developed by Wulff and Mata is a reasonable direction for future work.

The evidence from Wulff and Mata (2025) provides the strongest available empirical case for preferring transformer-based embeddings over LSA for this kind of application. The gap is large and consistent across multiple criteria and not a matter of theoretical preference. Transformer models have their own limitations, which we take up in the next section.

#### Validation against human judgments

The validation study tested whether ST5 similarity scores correspond to human semantic similarity judgments on a subset of 15 scale pairs drawn to represent the cluster structure identified in the *Semantic similarity analysis* section. The human benchmark was highly reliable, confirming that mean human ratings constitute a stable reference for model validation. At the individual level, rank correlations with the model were consistently positive and above zero, and inter-rater agreement was moderate, with considerable variability across rater pairs. Agreement was stronger at the extremes of the similarity distribution, suggesting that pairs ranked lowest and highest by the model tracked human rankings with greater fidelity than pairs in the intermediate range, where semantic distinctions are subtler. To our knowledge, this is one of the few studies to contrast model-based similarity judgments with human judgments within a psychometric context. Comparable approaches, such as the Semantic Scale Network (Rosenbusch et al., 2020) and the transformer-based analysis by Wulff and Mata (2025), did not include this kind of validation against human raters.

These results support the automated semantic analysis as a viable first-pass mapping tool that captures the broad structure of semantic relationships among subjective time scales in a manner consistent with human judgment. A key practical advantage is that this approach does not require collecting new empirical data to characterize relationships among measures, operates on existing scale content, and is scalable to large datasets that manual review alone could not cover within a reasonable timeframe.

These strengths come with boundary conditions worth acknowledging. The validation sample was small and recruited via convenience sampling, and a broader, more varied sample of raters would be needed to establish the generalizability of the human benchmark with confidence. The selection of the six scales for the validation task was guided by the ST5’s own clustering output, meaning the pairs evaluated were those the model had identified as representative of its structure. In contrast, pairs in the intermediate similarity range, where model and human judgments diverged more, were under-represented. These design choices were practical rather than arbitrary, but they mean the validation is strongest where the model performs best and more uncertain where it most needs scrutiny.

The ST5 also has limitations independent of the validation design. Because it was not fine-tuned on psychological scale items, its embeddings capture general semantic relationships rather than the theoretical distinctions that matter within a specific research tradition. Two scales can occupy similar regions of the embedding space while measuring constructs that a domain expert would treat as distinct, and the model has no basis for resolving that difference. The construct-item alignment results (*Pairwise similarity matrices and scale-construct alignment*) are instructive here: 47% of constructs showed greater similarity to items from another scale than to their own, a pattern the model identifies but cannot interpret.

Taken together, these considerations define the scope of the embeddings-based approach. It is a tool for mapping and screening that identifies candidate redundancies, reveals the broad organizational structure of a measurement domain, and does so without requiring new data collection. What it cannot do is settle questions that require theoretical judgment: whether similar scales are interchangeable for a given research purpose, whether a particular instrument suits a specific question, or whether a discrepancy between construct definition and item content signals a validity problem or a considered theoretical choice. Those questions are addressed in the following section.

### Practical recommendations

The recommendations below translate the findings from the section Cluster structure: Time perspective and temporal experience into practical guidance for instrument selection, as summarized in Table 2. The decision process follows two stages. In Stage 1, the researcher is directed to the appropriate cluster (Temporal Experience or Time Perspective) depending on whether the research question concerns how time is felt and perceived in a first-person sense or how individuals orient themselves across past, present, and future. In Stage 2, the appropriate subgroup and instrument are identified within each cluster.

**Table 2:** Summary of recommended subjective time scales

CLUSTER 1 – TEMPORAL EXPERIENCE (Is your primary interest in how time is felt, perceived, and conceptualized?)						
Scale	Primary construct	Alignment	*N*	Reliability	Items	Best suited for
*Temporal Metacognition*						
METP (Yu et al., 2023)	Metacognitive experience of time passing	✓	2,876	0.82	15	Experiential awareness of time passing
MQT (Lamotte et al., 2014)	Metacognitive knowledge of time distortions	✗	532	0.67	12	Explicit knowledge of factors distorting time perception; self vs. other comparison
TMQ (Sobol-Kwapinska & Nosal, 2009)	Symbolic and evaluative meaning of time	✗	251	0.79	95	Abstract or culturally mediated conceptualizations of time; discourse or mixed-methods research
*Time Pressure*						
CTPI (Denovan & Dagnall, 2019)	Chronic time pressure	✓	564	0.85	13	Chronic time pressure as a primary construct; heterogeneous adult samples
STQ – TP subscale (Wittmann & Lehnhoff, 2005)	Subjective time pressure	✗	499	0.78	5	Time pressure as one variable among many; brevity is required; lifespan research
TiME (Schiros et al., 2023)	Temporal processing	✗	215	0.69	18	Clinical (e.g., ADHD) and academic functioning contexts
*Other Instruments*						
TDS (Holman & Silver, 1998)	Temporal disintegration following trauma	✓	85	0.83	7	Acute stress, dissociation, psychological sequelae of trauma
TPS (Niiya, 2019)	Time as a social resource	✓	501	0.78	10	Interpersonal behavior, social obligation, prosocial motivation
CLUSTER 2 – TIME PERSPECTIVE (Is your primary interest in how individuals orient themselves toward past, present, and future?)						
Scale	Primary Construct	Alignment	N	Reliability	Items	Best suited for
*Multidimensional Time Perspective*						
TFS (Shipp et al., 2009)	Temporal frequency per frame	✓	1,671	0.74	12	Attentional deployment across temporal frames; large batteries where brevity matters
AATAS (Mello et al., 2016)	Temporal attitudes (affective valence per frame)	✗	388	0.81	30	Affective valence of past, present, and future; adolescent and lifespan research
BTPS (Webster, 2011)	Temporal integration (past-future motivation)	✓	238	0.88	28	Motivational integration of past and future; well-being and positive psychology research
ZTPI (Zimbardo & Boyd, 1999)	Temporal attitudes (broad; mixed content)	✗	1,034	0.74	56	Literature continuity; broad coverage of temporal attitudes and behaviors
TOS (Holman & Silver, 1998)	Temporal orientation	✗	72	0.73	28	Not recommended as a primary instrument
Time-Styles Scale (Usunier & Valette-Florence, 1994)	Time styles (scheduling, past, future, time pressure)	✗	300	0.70	29	Not recommended as a primary instrument
TAS (Rojas-Méndez et al., 2002)	Time attitudes (past, present, future)	✗	2,155	0.39	20	Not recommended as a primary instrument
*Future Time Perspective*						
CFC-14 (Strathman et al., 1994)	Future orientation (consequences of actions)	✓	993	0.80	12	General-purpose future orientation; decision-making and health behavior research
FTP Scale (Carstensen & Lang, 1996)	Future time perspective (remaining lifetime)	✗	329	0.86	10	SST framework; aging, health, and motivation research
Work Prospection Scale (Rutten et al., 2022)	Work-related future orientation	✓ [C]	1,024	0.71	12	Occupational and organizational research contexts
FTP-AYA (Lyu & Huang, 2016)	Future time perspective for youth	✓ [P]	1,480	0.66	28	Adolescent and young adult samples; lifespan research
Future Anxiety Scale (Zaleski, 1996)	Future affectivity (anxiety, dread)	✓ [C]	1,116	0.92	25	Negative affective engagement with the future; anxiety and stress research
MQFTP (Brothers et al., 2014)	Future time perspective (multidimensional)	✗	625	0.70	12	No clear advantage over CFC-14 on alignment or psychometric quality
PFOS (Lukwago et al., 2001)	Present and future orientation	✗	72	0.72	10	Small validation sample; insufficient psychometric evidence
FTP Scrambled Sentences Task (Biondolillo & Epstein, 2021)	Future time orientation (implicit measure)	✗	533	0.71	16	When self-report reactivity is a concern; note weaker alignment profile
*Other Instruments*						
OWBQ (Şimşek & Kocayörük, 2013)	Ontological well-being (life project evaluation)	✓	1,278	0.74	24	Evaluative engagement with life trajectory; well-being and narrative identity research
MTCQ (Morgenroth et al., 2021)	Metacognitive temporal coping strategies	✗	1,283	0.72	20	Emotion regulation; temporal reappraisal in coping research
PTOS (Sobol-Kwapinska, 2009)	Present orientation	✓	320	0.72	35	Present orientation as sole target construct; note high item cost
P-ETP (Vowinckel et al., 2017)	Present orientation	✓	151	0.88	10	Studies on balanced time perspective
TSWLS (Pavot et al., 1998)	Life satisfaction across time frames	✓	451	0.92	15	Developmental and lifespan studies examining evolving life satisfaction

Alignment indicates whether the instrument’s item content showed correct top-1 assignment in the construct-item alignment analysis (✓ = match; ✗ = non-match). [C] = contextual fit qualifier (instrument designed for a specific research context); [P] = population qualifier (instrument designed for a specific population with validation extended to others). *N* = sample size in the original validation study. Reliability = lower-bound reliability coefficient reported in the original validation study

Four criteria were used to guide selection: theoretical alignment between construct definition and operationalization, psychometric quality and validation evidence, practical cost, and fit with the target population and research question. The first two are the most important, while the remaining two serve as context-dependent tiebreakers. Table 2 summarizes how the instruments perform according to these criteria, and the following sections describe the reasoning behind each recommendation.

#### Choosing a measure from the temporal experience cluster

This cluster should be selected when the research question concerns how time is felt and experienced, rather than how individuals position themselves across temporal horizons. The cluster is diverse, as its instruments were built for different purposes and designed to target different facets of subjective time. The main challenge, therefore, is matching the instrument’s construct to the research question, rather than choosing among overlapping alternatives.

The cluster contains two main subgroups (temporal metacognition and time pressure), along with two instruments that do not fit into either. Within each subgroup, the alignment criterion points to one instrument: the Metacognitive Experience of Time Passing Scale for temporal metacognition (METP; Yu et al., 2023) and the CTPI for time pressure (Denovan & Dagnall, 2019). Both showed correct assignment in the construct-item alignment analysis, have the strongest psychometric profiles in their subgroups, and are grounded in theoretical frameworks that motivated their development. Specifically, the METP is based on Flavell’s (1979) account of metacognition and Flaherty’s (2018) model of time experience, while the CTPI builds on Szollos’s (2009) conceptual review of chronic time pressure. This combination of theoretical grounding, construct-item correspondence, and psychometric quality is what distinguishes them from alternatives within their respective subgroups.

*Temporal metacognition:* The case for the METP (Yu et al., 2023) begins with Flavell’s (1979) distinction between metacognitive knowledge, the target of the MQT (Lamotte et al., 2014), and metacognitive experience, the target of the METP (Yu et al., 2023). This distinction is the main reason to prefer the METP when the focus is on experience. Beyond this theoretical alignment, the METP also benefits from a qualitative item-development process, demonstrates measurement invariance, and shows adequate reliability. Its main limitation is cultural: it was developed with Chinese college students, and cross-cultural validation is needed before it can be confidently used in other populations.

The MQT is the better choice when metacognitive knowledge of temporal distortions is the construct of interest, or when comparing self-referential and other-referential beliefs is relevant. It is also a natural fit for experimental designs investigating whether beliefs about temporal distortions predict or moderate performance on behavioral timing tasks, a question of theoretical relevance given evidence that beliefs about temporal continuity between events can influence duration estimates (Glasauer & Shi, 2022). The TMQ (Sobol-Kwapinska & Nosal, 2009) is appropriate when the research question concerns symbolic or cultural conceptualizations of time rather than metacognitive awareness.

*Time pressure:* The case for the CTPI (Denovan & Dagnall, 2019) is more straightforward. It is the only instrument in the time pressure subgroup with a dedicated theoretical account of the construct, confirmed construct-item alignment, cross-sample measurement invariance, and adequate reliability. The STQ time pressure subscale (Wittmann & Lehnhoff, 2005) is a possible alternative when brevity is required. The TiME (Schiros et al., 2023), on the other hand, fits research on temporal processing more broadly, encompassing self-regulation, time estimation, and scheduling, but not time pressure as a standalone construct.

It is also worth noting a terminological issue. What Schiros et al. (2023) refer to as “temporal processing” concerns the subjective and behavioral management of time in daily life. This usage differs from the more restricted sense employed by Thönes and Stocker (2019), who define temporal processing as the ability to detect fundamental temporal information, such as the order and simultaneity of stimuli. Researchers should be mindful of this distinction when situating the TiME within the broader time perception literature.

##### Other instruments

The TDS (Holman & Silver, 1998) and the TPS (Niiya, 2019) fall outside the main subgroups. Both showed alignment between constructs and items and covered constructs with no equivalents elsewhere in the review. The TDS is suited for trauma and dissociation research, while the TPS fits work on time as a social resource in interpersonal and prosocial contexts. Neither has an extensive psychometric record, however, which should be weighed against their confirmed construct-item correspondence.

#### Choosing a measure from the time perspective cluster

This cluster should be selected when the research question concerns how individuals orient toward, evaluate, or distribute attention across temporal frames. Compared to the Temporal Experience cluster, this one requires more care. It is the most redundancy-prone part of the review, and “time perspective” covers more than one construct. Mello and Worrell (2015) distinguish at least five dimensions: attitude, orientation, frequency, relation, and meaning. Most measurement problems in this cluster can be traced to instruments that operationalize one dimension while claiming to measure another. The alignment results indicate that several instruments showed higher semantic similarity between their construct definitions and another instrument’s items than between their own construct definitions and their items. This does not necessarily mean the instruments are invalid, but it does reveal which part of the construct each instrument actually captures and flags potentially problematic cases.

##### Multidimensional instruments

The practical implication is to start with the dimension question. Table 2 maps each multidimensional instrument to the Mello and Worrell (2015) dimension it captures most directly. For attentional deployment across temporal frames, the TFS (Shipp et al., 2009) is the clearest choice, as its items track the core semantic content of time perspective definitions and, at 12 items, is also the most practical option in this group. For the affective valence of temporal frames, including negative poles, the AATAS (Mello et al., 2016) is the most appropriate instrument. It is also the only one in this group designed for adolescent samples, with validation subsequently extended to adults. For research on the motivational integration of past and future in well-being contexts, the BTPS (Webster, 2011) has the strongest reliability in the group and good construct-item alignment. However, its positive-affect focus is a real constraint when negative temporal engagement is relevant.

The ZTPI (Zimbardo & Boyd, 1999) is a separate case. It is often considered the first standardized psychometric instrument for measuring time perspective (Stolarski et al., 2018), and researchers working in that tradition will often find it difficult to avoid, as most subsequent instruments were developed in dialogue with it. Our analysis found that its construct definition aligns more closely with TFS items than with its own, suggesting that its operationalization extends beyond the core definitional content toward a broader, more heterogeneous set of facets. Beyond our own findings, the literature documents additional concerns regarding the ZTPI’s factor structure variability across cultural and linguistic contexts (Temple et al., 2019), the inconsistent psychometric properties among its versions (Davis & Ortiz, 2017; Perry et al., 2020), and its mixing of temporal attitudes with behavioral and personality-adjacent content in ways that complicate construct-specific interpretation (Mohammed & Marhefka, 2019). Our recommendation is to use the ZTPI when continuity with the existing literature is the priority, while treating it as a starting point rather than a standard above scrutiny.

Among the remaining instruments, the TAS (Rojas-Méndez et al., 2002) may warrant caution as a primary measure, given that its lower-bound reliability in the original validation study was 0.39. The TOS (Holman & Silver, 1998) was validated in a relatively modest sample of 72 participants, suggesting that its psychometric properties would benefit from further corroboration. The Time-Styles Scale (Usunier & Valette-Florence, 1994) shares considerable conceptual ground with the Time Pressure subgroup, and researchers interested in its content may find that the instruments in that subgroup offer more refined alternatives.

##### Future time perspective

The FTP subgroup contains the strongest case of micro-level redundancy in the review. Eight instruments share the label, yet their item content, theoretical grounding, and empirical behavior diverge in several ways. Two issues deserve attention before selecting among them.

The first is whether FTP is itself a unitary construct. Kooij et al.’s (2018) meta-analysis proposed that FTP encompasses at least three distinct dimensions: future orientation (the extent to which individuals focus on and plan for the future), continuity (the perception that one’s future extends from and connects to the present), and affectivity (emotional responses to the future, including hope and anxiety). No instrument in this review covers all three simultaneously. This is consequential because the choice of instrument commits the researcher to a particular operationalization, and findings may not generalize across operationalizations. Kooij et al. (2018) illustrated this point by showing that age was negatively related to FTP when measured with the FTP Scale (Carstensen & Lang, 1996), but positively related when measured with the ZTPI Future subscale. In other words, the same variable can lead to opposite conclusions depending on which facet is captured.

The second issue is construct-item alignment. Four of the eight instruments showed the correct construct-item alignment: the CFC-14 (Strathman et al., 1994), the Work Prospection Scale (Rutten et al., 2022), the FTP Scale for Adolescents and Young Adults (FTP-AYA; Lyu & Huang, 2016), and the Future Anxiety Scale (Zaleski, 1996). The remaining four did not, suggesting that these instruments operationalize FTP through facets that diverge from the core semantic content of their definitions. Three of the four non-matching instruments, the FTP Scale (Carstensen & Lang, 1996), the Multidimensional Questionnaire of FTP (Brothers et al., 2014), and the Present and Future Orientation Scales (Lukwago et al., 2001), are among the broader operationalizations in the group, consistent with the pattern observed in the multidimensional subgroup.

Selection among these instruments should center on two questions: which FTP dimension is relevant to the research question, and does the research context or population impose additional constraints?

For general-purpose research on future orientation, the CFC-14 (Strathman et al., 1994) is the preferred choice. It showed good construct-item alignment, has a moderate validation sample, good reliability, and a low item count. It operationalizes FTP as the tendency to consider the future consequences of present actions, mapping onto the future orientation dimension of Kooij et al.’s (2018) framework. The FTP Scale (Carstensen & Lang, 1996) is the main alternative for researchers working within Socioemotional Selectivity Theory, in which FTP is defined as the perception of remaining time in life and its motivational consequences. It did not show good construct-item alignment, but it has the strongest reliability among the general-purpose instruments, with only ten items and an extensive empirical record in aging and health research. Researchers outside this theoretical tradition should note that it operationalizes the continuity dimension of Kooij et al.’s framework rather than future orientation broadly. The Multidimensional Questionnaire of FTP (Brothers et al., 2014) and the Present and Future Orientation Scales (Lukwago et al., 2001) offer no clear advantage over the CFC-14 in terms of construct-item alignment, psychometric quality, or item cost.

For researchers with a specific context or population in mind, the more specialized instruments are appropriate. The Work Prospection Scale (Rutten et al., 2022) had good construct-item alignment and was designed for organizational contexts, whereas FTP concerns the anticipation and planning of work-related futures. Its large validation sample and parsimonious item count make it a practical option for occupational research, though its reliability is acceptable rather than strong. The FTP-AYA (Lyu & Huang, 2016) showed good construct-item alignment and was developed with younger populations in mind, with validation extended across the lifespan. It has the largest validation sample in the group, but its reliability is the lowest, and its 28 items represent the highest item cost among the specialized instruments. The Future Anxiety Scale (Zaleski, 1996) showed good construct-item alignment and the strongest reliability in the subgroup. Still, it operationalizes the affectivity dimension of FTP (i.e., emotional responses to the future, including worry and dread) rather than future orientation or continuity. It is best suited for research in which negative affective engagement with the future is the primary construct, not FTP as a cognitive or motivational orientation.

The Scrambled Sentences Task (Biondolillo & Epstein, 2021) stands apart from the others. It employs a behavioral task format in which respondents arrange scrambled words into sentences, with future-oriented versus present-oriented constructions serving as the outcome measure. This is valuable when self-report bias or demand characteristics are a concern, but the instrument failed to show good construct-item alignment, and its reliability is moderate rather than strong. Researchers should balance its methodological distinctiveness against its weaker alignment.

The practical recommendation is to clarify which FTP dimension is most relevant. If future orientation as attentional and motivational engagement is central, choose the CFC-14. For research rooted in SST and focused on the perception of remaining lifetime, select the FTP Scale. For context- or population-specific questions, use the Work Prospection Scale, the FTP-AYA, or the Future Anxiety Scale, as appropriate. The remaining instruments offer no clear advantage that would justify their selection over these alternatives.

It is worth noting, however, that none of the instruments reviewed here, and to our knowledge none currently available, covers the three dimensions proposed by Kooij et al. (2018) within a single unified measure. Future orientation, continuity, and affectivity remain operationalized in separate instruments, each capturing a different facet of the construct. Developing an instrument that integrates these dimensions while maintaining construct clarity and psychometric quality remains an open challenge for the field.

##### Other instruments

The Ontological Well-Being Questionnaire (OWBQ; Şimşek & Kocayörük, 2013), the TSWLS (Pavot et al., 1998), and the MTCQ (Morgenroth et al., 2021) are placed in this cluster due to semantic proximity rather than construct equivalence. The OWBQ uses temporal frames to organize the affective evaluation of the life project, and the TSWLS assesses life satisfaction by dividing it into past, present, and future time frames. In this sense, they are well-being instruments structured around time, rather than time perspective measures in a strict sense. The MTCQ, on the other hand, assesses coping strategies that draw on temporal reappraisal and, conceptually, aligns more closely with the temporal metacognition subgroup within the Temporal Experience cluster. All three fit their intended research contexts, but none fit the time perspective construct well.

It is worth noting, however, that all of these scales exhibited good construct-item alignment. For this reason, we recommend that their use be guided by the specific research context. They are unique tools and should be adopted when they help answer theoretically well-defined questions, such as exploring the temporal dynamics of well-being or specific temporal coping mechanisms, but they should not be selected as general proxies for time perspective.

While the preceding scales use time frames to evaluate other psychological constructs, two instruments refocus on time perspective itself by targeting the present. Both offer an adaptive alternative to traditional operationalizations, such as the ZTPI’s Present-Hedonistic subscale. The P-ETP scale (Vowinckel et al., 2017) was developed to complement the past and future subscales of the BTPS (Webster, 2011) by aligning a positive present-time perspective with concepts of mindfulness and flow. As a ten-item instrument, it imposes a low practical cost. The PTOS (Sobol-Kwapinska, 2009) offers another operationalization of the present perspective, emphasizing the ability to discern the unique value of each moment. It was developed to differentiate a mindful, active focus from both hedonistic and fatalistic present orientations. However, at 35 items, it represents a high practical cost for evaluating a single time frame.

While both scales are conceptually well suited for research that contrasts adaptive, active present engagement with maladaptive or pleasure-driven present orientations, their adoption requires careful consideration. The psychometric properties reported in the original validation studies for both instruments are insufficient to establish their structural quality or generalizability. We therefore recommend that the use of either scale be conditioned on a clear definition of the construct of interest, preferably one that aligns with the respective authors’ theoretical frameworks, and on the specific demands of the research context.

## Conclusions

Thirty self-report instruments for measuring subjective time are not necessarily too many. A domain spanning temporal metacognition, time pressure, the disintegration of temporal experience, time-related traits, and the symbolic meaning individuals attach to time needs a comprehensive measurement toolkit. The problem this study identified is not proliferation per se, but instruments accumulating without a principled account of how they differ, definitions moving away from the items written to operationalize them, and researchers selecting among options that appear distinct by label but converge in content.

Hierarchical clustering of the full similarity matrix reveals two broad clusters: Temporal Experience and Time Perspective. Each cluster includes internal subgroups that vary in redundancy and construct coherence. The construct-item alignment analysis found that nearly half of the instruments in this review are not aligned most closely with their own definitions. This figure is striking even when considering the expected difference between abstract constructs and concrete items. This pattern suggests a measurement collection that has grown faster than the conceptual framework needed to organize it.

The boundary between Temporal Experience and Time Perspective does not map cleanly onto theoretical taxonomies from experimental studies. Instead, it shows how researchers have operationalized their questions across different traditions. One tradition asks how time feels and is structured in immediate experience. Another asks how individuals position themselves across past, present, and future horizons. The fact that this boundary emerges from the data rather than being imposed on it suggests that it captures something meaningful about how subjective time is studied in practice. One of the main challenges is narrowing the gap between how the field measures subjective time and how it theorizes it.

The practical recommendations outlined are a step in that direction. They work as an attempt to make the selection process more deliberate: anchored in what constructs are actually defined to measure, evaluated against what has been empirically established, and sensitive to the costs and constraints of real research contexts. The goal is not to reduce the measurement toolkit to a single preferred instrument per construct, but to improve instrument choice, moving selection from convenience toward construct alignment and evidence.

The method itself is replicable, scalable, and applicable to other measurement domains. Wherever constructs have proliferated and instruments have accumulated, a similar approach can provide a first characterization. This involves embedding items and definitions independently, clustering the resulting similarity matrix, and analyzing the correspondence between them. Although this should not replace theoretical judgment or empirical validation, it can improve these processes by highlighting where scrutiny is needed and where existing instruments already perform well.

Future work can proceed along several lines. Domain-specific fine-tuning of a language model on psychological scale items can improve the precision of semantic similarity estimates and allow the method to recover theoretical distinctions that a general-purpose model cannot. Applying the construct-item alignment analysis to empirical validity data, rather than relying solely on semantic similarity, would allow direct testing of which misalignments between definitions and items produce validity problems. Extending the analysis to other measurement domains can test whether the two-cluster structure observed here is specific to subjective time or reflects a more general pattern in how psychological constructs are organized across adjacent literatures. Furthermore, the map produced here could inform the development of *synthesis instruments* (i.e., measures designed with explicit awareness of the existing toolkit), sampling the construct space more deliberately than instruments developed in isolation. Rather than adding to an already crowded toolkit, such instruments would consolidate what has been learned, covering the dimensions identified by prior work while eliminating redundant content and resolving the construct-item misalignments documented here. The case for a unified FTP measure integrating Kooij et al.’s (2018) three dimensions is the most concrete example, but the same logic applies wherever the review identified theoretical coherence without a corresponding measurement solution.

## Data Availability

Supplementary material with complete data descriptions is available on the Open Science Framework (OSF) at https://osf.io/rj3kx/.

## Footnotes

1 We thank an anonymous reviewer for suggesting the post hoc experiment.

## Code availability

All analysis scripts are available on GitHub at https://github.com/Rodrigo-Motta/Timing_LLM.
